# Universal genome-wide association studies: Powerful joint ancestry and association testing

**DOI:** 10.1016/j.xhgg.2023.100235

**Published:** 2023-08-30

**Authors:** Daniel Shriner, Amy R. Bentley, Mateus H. Gouveia, Elisabeth F. Heuston, Ayo P. Doumatey, Guanjie Chen, Jie Zhou, Adebowale Adeyemo, Charles N. Rotimi

**Affiliations:** 1 Center for Research on Genomics and Global Health, National Human Genome Research Institute, Bethesda, MD 20892, USA

## Abstract

The vast majority of human populations and individuals have mixed ancestry. Consequently, adjustment for locus-specific ancestry is essential for genetic association studies. To empower association studies for all populations, it is necessary to integrate effects of locus-specific ancestry and genotype. We developed a joint test of ancestry and association that can be performed with summary statistics, is independent of study design, can take advantage of locus-specific ancestry effects to boost power in association testing, and can utilize association effects to fine map admixture peaks. We illustrate the test using the association between serum triglycerides and *LPL*. By combining data from African Americans, European Americans, and West Africans, we identify three conditionally independent variants with varying amounts of ancestrally differentiated allele frequencies. Using out-of-sample data, we demonstrate improved prediction achievable by accounting for multiple causal variants and locus-specific ancestry effects at a single locus.

## Teaser

We describe a joint test of ancestry and association calculated from summary statistics from admixture mapping and association studies. Using data for serum triglycerides and *LPL* from African Americans, European Americans, and West Africans, we identify three conditionally independent variants with varying amounts of ancestrally differentiated allele frequencies.

## Introduction

The process of admixture refers to interbreeding between previously isolated populations and results in individuals with mixed ancestry. The idea of admixture mapping as a gene mapping tool was described in 1954^1^ but did not become practical at the genome-wide scale until the early 2000s with the accumulation of marker data^2^ and the development of methods.^3^ The use of mixed ancestry to map trait genes across the genome was first possible for African Americans,^2^^,^^3^ although it is now recognized that the overwhelming majority of human populations and individuals have mixed ancestry.^4^^,^^5^^,^^6^ In a chromosomal region containing an allele that increases risk or a trait value, there should be an excess of ancestry from whichever parental population has a higher frequency of the risk or trait-increasing allele at that locus.^3^ Admixture mapping is based on correlating allele frequency differences between parental populations with the phenotype, which is the same information that determines whether population stratification is a confounder in genetic association studies.

**Figure 1:**
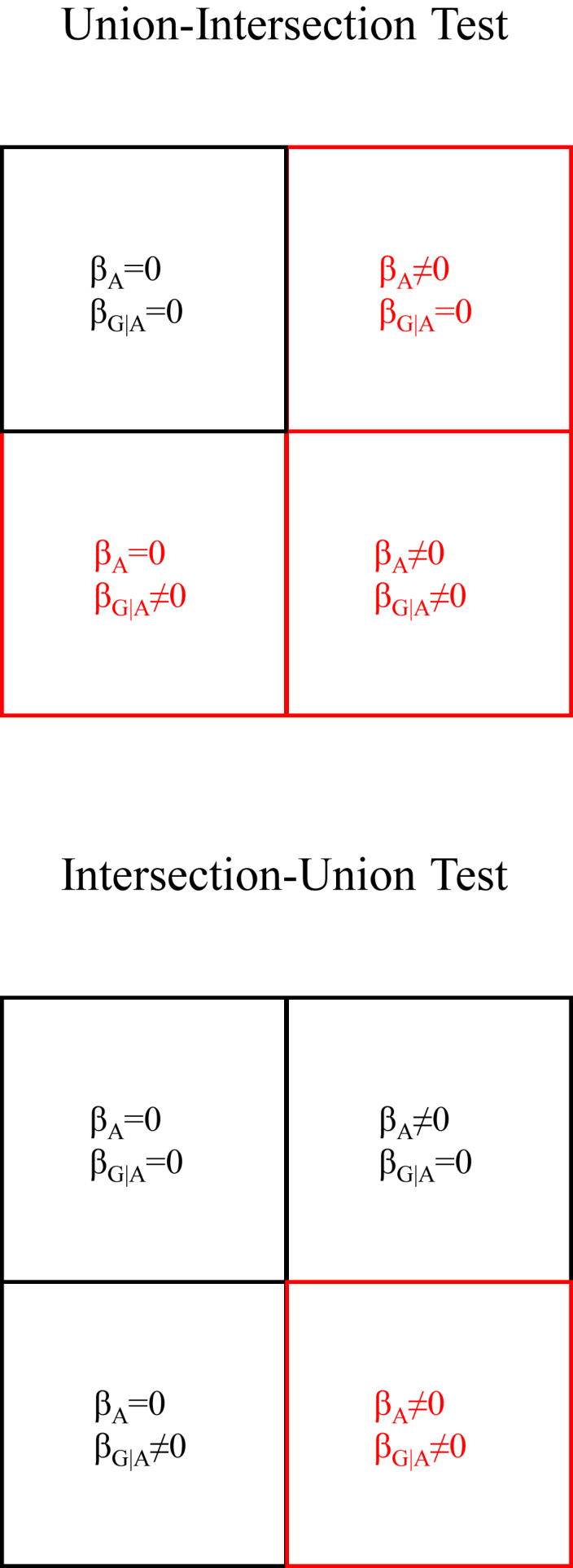
Contrasting design of the union-intersection and intersection-union tests β_A_ denotes the effect of locus-specific ancestry, and β_G|A_ denotes the effects of genotype conditional on locus-specific ancestry. Black represents composite null hypotheses, and red represents composite alternative hypotheses.

A union-intersection test (UIT) involves the composite null hypothesis that all null hypotheses are true against the composite alternative hypothesis that at least one alternative hypothesis is true.^7^^,^^8^ In the gene mapping context, the composite alternative hypothesis is that (1) there is a locus-specific ancestry effect but no association effect, (2) there is an association effect but no locus-specific ancestry effect, or (3) there are both locus-specific ancestry and association effects (Figure 1). The alternative hypothesis of a locus-specific ancestry effect but no association effect is tantamount to linkage without association and is typically not of interest. The alternative hypothesis of an association effect but no locus-specific ancestry effect is more powerfully tested using a standard association test. UITs are readily constructed by taking the sums of test statistics. Several UITs that are joint ancestry or association tests have been described for family data^9^ or population data.^10^^,^^11^^,^^12^^,^^13^ This class of tests includes the SUM test^10^ and the test implemented in Tractor.^13^ Tests that are based on $\chi^{2}$ statistics ignore the information contained in the signs of the association and locus-specific ancestry effects. Consequently, a test based on $\chi^{2}$ statistics could yield a false positive result if there is an association between increased risk or a trait value and an excess of ancestry from whichever parental population has a lower frequency of the risk or trait-increasing allele at that locus.

An intersection-union test (IUT) involves a composite null hypothesis that at least one null hypothesis is true against the composite alternative hypothesis that all alternative hypotheses are true.^7^^,^^8^ In the gene mapping context, the composite alternative hypothesis is that there are both locus-specific ancestry and association effects (Figure 1). A well-known example of an IUT in gene mapping is the transmission-disequilibrium test, which is a joint test of linkage and association and is based on the product of linkage and linkage disequilibrium.^14^ A second example is the MIX test,^10^ which is a product of locus-specific ancestry and association effects but is defined solely for the case-only design.^3^^,^^15^^,^^16^ The case-only design is not robust to misspecification of parental allele frequencies, and controls are necessary to address the possibility that cases and controls might have different ancestry proportions on average. A third example is the BMIX test,^17^ which suffers from the same loss of information as other tests based on $\chi^{2}$ statistics.

Here, we derive an IUT that is a joint ancestry and association test. This test is applicable for any study design and phenotype that can be analyzed using a generalized linear mixed model. Furthermore, this test is based on signed z statistics rather than $\chi^{2}$ statistics and so retains validity across all configurations of locus-specific ancestry and genotype states. We illustrate the utility of this test by untangling locus-specific ancestry and association effects at *LPL* (MIM: 609708) for serum triglycerides.^18^^,^^19^

## Materials and methods

### IUT

At a marker, let $z_{A}$ represent the *Z* score for a locus-specific ancestry effect and let $z_{G|A}$ represent the *Z* score for an association effect of genotype conditional on locus-specific ancestry. The product of these two *Z* scores is the test statistic for the joint test of ancestry and association. The product can only be nonzero if both *Z* scores are nonzero. The product follows the product normal distribution. We used the R package RMediation^20^ to evaluate p values and quantiles from the product normal distribution.

The signs of the *Z* scores convey useful information that would be lost in a test based on $\chi^{2}$ statistics. To illustrate, consider two-way admixed individuals. Assuming no parent-of-origin effects, there are three strata based on locus-specific ancestry, two homozygous and one heterozygous. Let locus-specific ancestry be coded as a categorical variable based on the number of alleles inherited from a specified parental population: 0 (homozygous for one ancestry), 1 (heterozygous), or 2 (homozygous for the other ancestry). Let genotype be coded as 0, 1, or 2 copies of the effect allele. Suppose that the locus-specific ancestry effect is positive. Then, that locus-specific ancestry effect can be explained by a positive association effect if the frequency of the effect allele is positively correlated with locus-specific ancestry or by a negative association effect if the frequency of the effect allele is negatively correlated with locus-specific ancestry. Under the assumption that the effect of allelic substitution is independent of background, a positive product constitutes evidence for joint ancestry and association, whereas a negative product does not. For this reason, the product test is one tailed. To operationalize this logic, let $f_{0}$ be the frequency of the effect allele in the stratum of locus-specific ancestry coded 0 and let $f_{2}$ be the frequency of the effect allele in the stratum of locus-specific ancestry coded 2. The test statistic is defined as $\text{sign}\left(f_{2}-f_{0}\right)Z_{A}Z_{G|A}$. This test is designed for pairwise comparisons assuming at least two ancestral strata.

### Population descriptors

The definition of admixture as the process of intermating between previously isolated populations involves, at a minimum, three populations (an admixed population and two parental or source populations) and two timescales. In our study, the descriptor African American refers to descendants of the Middle Passage. Admixed African Americans resulted from intermating over the last few hundred years. The descriptors African and European refer to the two primary source populations. These two descriptors are intentionally broad and inclusive in order to reflect the genetic diversity in both sources. The timescale of isolation encompasses the tens to hundreds of thousands of years since the split of anatomically modern humans into lineages that predated human dispersal out of Africa.

### Studies

We integrated individual-level data from eight genetic epidemiology studies comprising 23,643 African Americans (Table S1): the Atherosclerosis Risk in Communities (ARIC) study,^21^ the Cleveland Family Study (CFS),^22^ the Genetic Epidemiology Network of Arteriopathy (GENOA),^23^ the Howard University Family Study (HUFS),^24^ the Jackson Heart Study (JHS),^25^ the Multi-Ethnic Study of Atherosclerosis (MESA),^26^ the Sea Islands Genetic Network (SIGNET),^27^ and the Women’s Health Initiative (WHI).^28^ We integrated individual-level data from five genetic epidemiology studies comprising 17,684 European Americans (Table S1): ARIC,^21^ the Coronary Artery Risk Development in Young Adults (CARDIA) study,^29^ the Framingham Heart Study (FHS),^30^ GENOA,^23^ and MESA.^26^ We also analyzed individual-level data for 3,583 West Africans from Ghana or Nigeria (Table S1) from the Africa America Diabetes Mellitus (AADM) study.^31^ Ethical approval was obtained from the National Institutes of Health and from the ethical committees in each study site. All participants gave written informed consent.

### Phenotype and covariates

Serum triglyceride (mg/dL) measurements for HUFS and AADM were made on fasting samples and determined enzymatically, as previously reported.^19^^,^^31^ For those studies accessed through dbGaP, data were extracted from pht000114 (ARIC), pht001588 (CARDIA), pht001902 (CFS), pht006027 (FHS), pht006655 (GENOA), pht008729 (JHS), pht001116 (MESA), pht002436 (SIGNET), and pht003419 (WHI). Data for serum triglycerides were cleaned based on inspection of the distribution within each study separately to remove outliers and then natural-log transformed. Age was extracted for the visit corresponding to the serum triglyceride measurement.

### Genotype cleaning and imputation

For each study, we followed the same genotype cleaning and imputation steps. First, markers that were strand ambiguous or monomorphic were removed. Using PLINK, we then filtered for individual-level missingness of 0.1, per-marker missingness of 0.05, and a Hardy-Weinberg equilibrium p value of $1\times 10^{-10}$. We used the liftover tool to migrate coordinates from NCBI/B36 to GRCh37. We then used the bcftools plugin fixref to orient all alleles as reference and alternate according to the file human_g1k_v37.fasta. Next, we used the Python script checkVCF.py to clean the datasets prior to imputation with the TOPMed server and the r2 reference panel (coordinates based on GRCh38). Imputed data were filtered based on an rsq quality score of 0.3 and a minor allele frequency of 0.5%. Imputed datasets were merged using bcftools.

### Inference of locus-specific ancestry

For each of the eight studies of African Americans, we inferred locus-specific ancestry using RFMix.^32^ We constructed a five-way reference panel based on a subset of 2,504 individuals in phase 3 of the 1000 Genomes Project^5^ and individual admixture proportions.^6^ Population identifiers as defined by the 1000 Genomes Project^5^ are reproduced in Table S2. From the AFR metapopulation, we retained 471 individuals (34 ACB, 1 ASW, 93 ESN, 87 GWD, 77 LWK, 76 MSL, and 103 YRI) with predominantly Central African, Eastern African, Omotic, Southern African, West-Central African, or Western African ancestry. From the AMR metapopulation, we retained 23 individuals (20 PEL and 3 MXL) with predominantly Amerindian or Circumpolar ancestry. From the EAS metapopulation, we retained 316 individuals (38 CDX, 85 CHB, 97 CHS, 62 JPT, and 34 KHV) with predominantly Japanese, Sino-Tibetan, or Southeastern Asian ancestry. From the EUR metapopulation, we retained 136 individuals (51 IBS, 31 FIN, 12 CEU, 9 GBR, and 33 TSI) with predominantly Arabian, Northern African, Northern European, Southern European, or Western Asian ancestry. From the SAS metapopulation, we retained 67 individuals (24 GIH, 21 ITU, 4 PJL, and 18 STU) with predominantly South Indian or Southern Asian ancestry. We ran RFMix with the settings -n 5 and -w 0.1. By inferring locus-specific ancestry within the reference panel, instead of treating the reference panel as fixed, we estimated 99.15% reliability of assignment of locus-specific ancestry (Table S3). We inferred locus-specific ancestry for each of the eight studies separately (Table S4) and then merged the calls across the intersection of markers that were genotyped in all eight studies. Calls were recoded as 0, 1, or 2 alleles inherited from the African parental population at the locus.

### Population structure

Principal-component analysis of the African data revealed that the top principal component separated Ghanaians from Nigerians.^33^ Principal-component analysis of the European data revealed that the top principal component reflected a north-to-south cline.^34^ Principal-component analysis of the African American data revealed that the first principal component correlated with the individual proportion of African ancestry (Figure S1). None of the top principal components correlated with study or genotyping array.

### Admixture mapping

To test the effect of locus-specific ancestry on phenotype, we regressed the phenotype on locus-specific ancestry using a linear mixed model, adjusted for age, sex, and study as fixed effects and the relatedness matrix estimated from locus-specific ancestry as a random effect (which simultaneously controls genome-wide ancestry, known relatedness, and cryptic relatedness). To reduce confounding by shared environmental effects among close relatives, unrelated individuals were extracted using --grm-cutoff 0.046875 (i.e., between 4^th^ and 5^th^ degree relatives) in GCTA.^35^ To perform the regression, we used the --mlma option in GCTA, which leaves out the chromosome being tested from the relatedness matrix. Note that this test does not require the phenotype to be stratified across ancestries or for there to be a significant effect of genome-wide ancestry.

### Structured association testing

To test the effect of genotype on phenotype, we regressed the phenotype on imputed dosages using a linear mixed model, adjusted for age, sex, and study as fixed effects and the genetic relatedness matrix as a random effect. Unrelated individuals were extracted using --grm-cutoff 0.046875 in GCTA. We used the Bioconductor package GENESIS, leaving out the chromosome being tested from the genetic relatedness matrix. To account for admixture in African Americans, we stratified the association using three strata of locus-specific ancestry (i.e., homozygous African, heterozygous, and homozygous European), an approach robust to the unknown genetic mode of inheritance.^36^^,^^37^ We combined the three sets of results across strata using the standard error scheme in METAL. Given that the genome-wide testing burden for association testing exceeds the genome-wide testing burden for admixture mapping in admixed African Americans by three orders of magnitude,^17^ the genome-wide significance level was set at $5\times 10^{-8}$. Annotation was retrieved from Ensembl release 105.^38^

### Genetic score and prediction

We generated a genetic score accounting for the effects of age, sex, and genetic variants. For each genetic variant, we weighted the allelic dosage by the estimated allelic effect size. We used the nonoverlapping set of individuals excluded upon filtering for relatedness.

## Results

**Figure 2:**
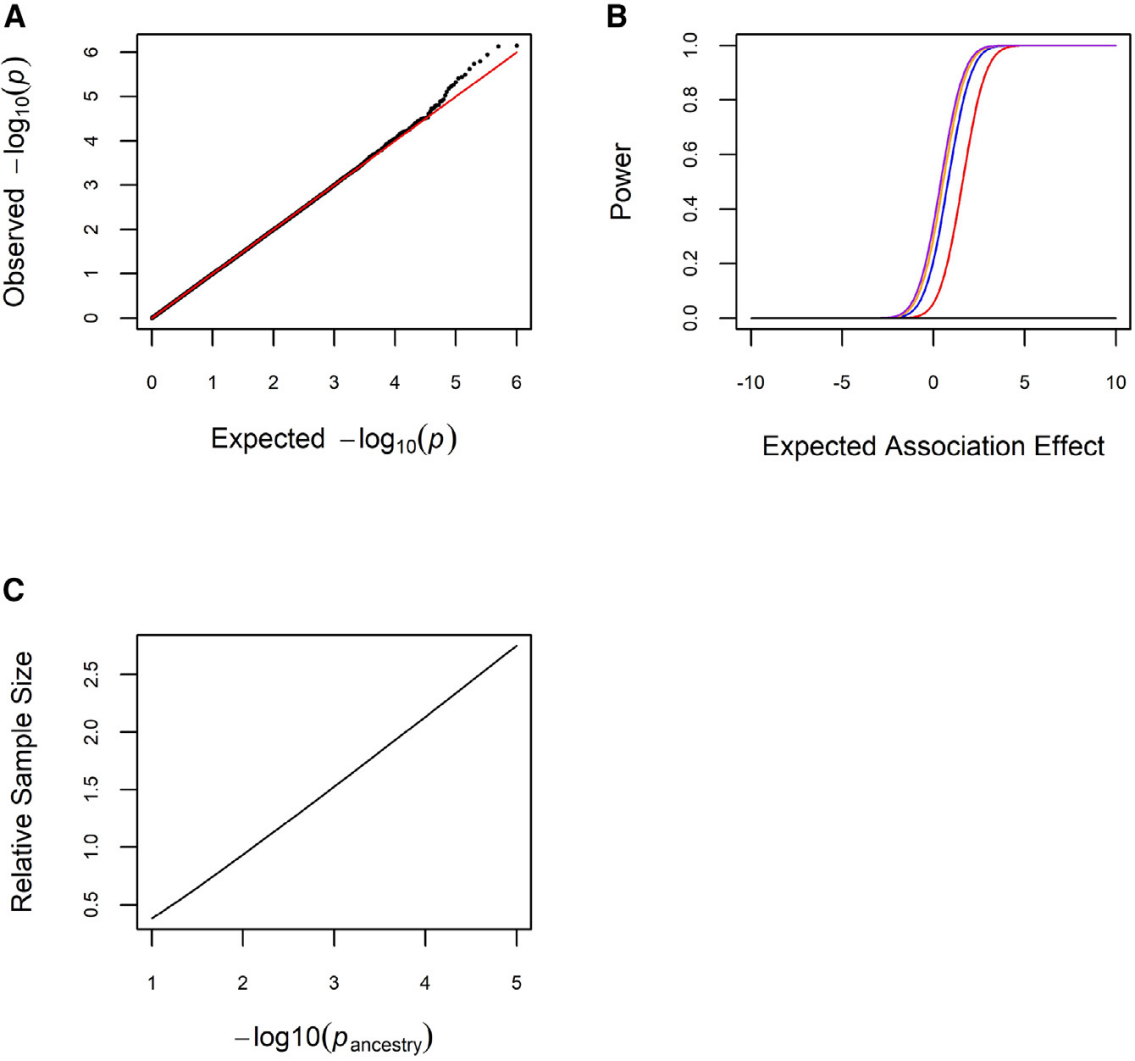
Performance of the joint ancestry and association test (A) Type I error rate. The black line represents data simulated under the null hypothesis, and the red line represents the expected uniform distribution of p values under the null hypothesis. (B) Power. We simulated the expected association effect as the mean of a normal distribution with unit variance and a realized locus-specific ancestry effect as a standardized *Z* score (black represents $Z_{A}=0$, red represents $Z_{A}=1$, blue represents $Z_{A}=2$, orange represents $Z_{A}=3$, and purple represents $Z_{A}=4$). The locus-specific ancestry and association effects are statistically independent. (C) Benchmarking. The x axis indicates $-\log_{10}\left(p_{ancestry}\right)$, and the y axis indicates the sample size of the association test required to achieve genome-wide significance by the standard association test relative to the joint test.

To integrate locus-specific ancestry and association effects, we derived an IUT to specifically test the composite alternative hypothesis of both locus-specific ancestry and association effects. Let $Z_{A}$ represent the *Z* score for a locus-specific ancestry effect and let $Z_{G|A}$ represent the *Z* score for an association effect of genotype conditional on locus-specific ancestry. The product of these two *Z* scores is a test statistic for the joint test of ancestry and association. Under the null hypothesis, the test statistic is distributed as the product of two standard normal random variables. Conditioning association testing on locus-specific ancestry leads to the property that the two random variables are conditionally independent. By incorporating ancestral allele frequencies into the test, we constrain the test to be one tailed. To establish the validity of this test, we generated 1,000,000 statistical replicates of three independent random variates from the standard normal distribution and calculated the test statistic by multiplying the sign of the first random variate by the product of the other two random variates. For a significance level α=0.05, the type I error rate was 0.049779, indicating that the one-tailed product test was valid (Figure 2A). On an HP EliteBook 830 G8 Notebook with a single Intel i7 processor running at 3 GHz, runtime for 1,000,000 tests was 112 s. As expected, for a given locus-specific ancestry effect, power increased as the association effect increased (Figure 2B). Also as expected, for a given association effect, power increased as the locus-specific ancestry effect increased (Figure 2B).

We next benchmarked the joint ancestry and association test relative to the standard association test (Figure 2C). We performed this benchmarking analytically (i.e., without simulations) by working directly with Z statistics and p values. Specifically, $p_{association}=5\times 10^{-8}$ corresponds to $Z_{G|A}=5.45$ and $p_{joint}=5\times 10^{-8}$ corresponds to $Z_{A}Z_{G|A}=14.52$. To achieve $p_{joint}=5\times 10^{-8}$ given a genome-wide significant admixture mapping signal with $p_{ancestry}=1\times 10^{-5}$ ($Z_{A}=4.42$), we require $Z_{G|A}=3.29$, corresponding to $p_{association}=1.01\times 10^{-3}$ and reflecting an increase in power equivalent to a 2.75-fold larger sample size to detect association relative to the standard association test. To achieve $p_{joint}=5\times 10^{-8}$ given a genome-wide association signal with $p_{association}=5\times 10^{-8}$, we require $p_{ancestry}=7.74\times 10^{-3}$, indicating a loss of power to detect association relative to the standard association test if the locus-specific ancestry effect is small.

Given prior evidence for a locus-specific ancestry effect at *LPL* for serum triglycerides,^19^ we estimated the size of this effect in 14,895 unrelated African Americans. Admixture mapping revealed an interval of 184.6 kb (from rs7821631 [NC_000008.11:g.19873864T>C] to rs13263007 [NC_000008.11:g.20058493C>T]), at which an increasing amount of European ancestry was associated with increased serum triglycerides ($Z=2.76,p=5.83\times 10^{-3}$). *LPL* is the only gene in this interval.

We next tested the association of genotype on serum triglycerides at all 1,679 variants in the 184.6 kb interval in 14,895 unrelated African Americans (Table S5). A total of 103 variants were genome-wide significant. When we required directional consistency with the locus-specific ancestry effect, 49 variants were genome-wide significant, reflecting fine mapping of the locus-specific ancestry effect. The joint test yielded an increase of genome-wide significant variants from 49 to 66. All 17 additional variants had association p values below genome-wide significance (p values ranged from $1.39\times 10^{-7}$ to $6.17\times 10^{-8}$). The increase in the number of variants genome-wide significant by the joint test compared with the standard association test reflects a gain in power.

**Figure 3:**
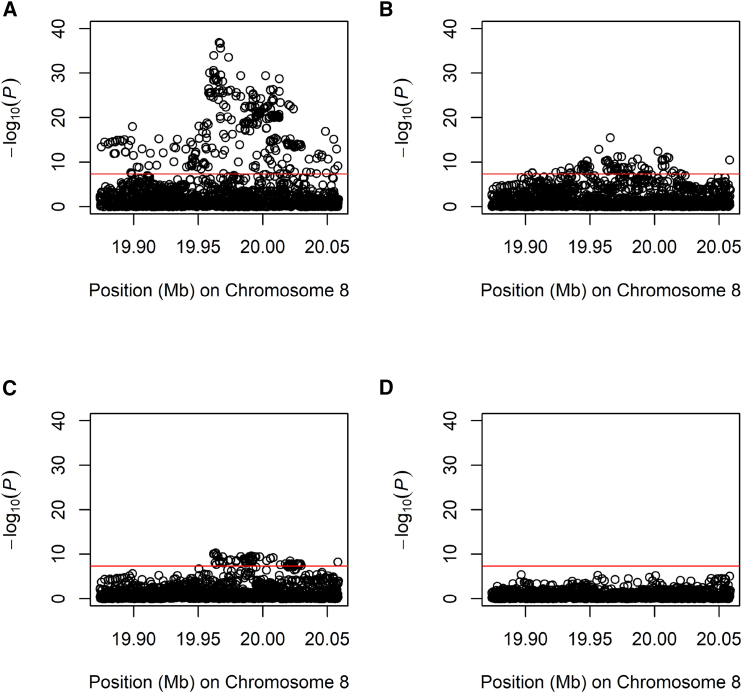
Meta-analysis of association results for Africans, African Americans, and Europeans (A) Unconditional analysis. (B) Conditional on rs3208305. (C) Conditional on rs3208305 and rs3289. (D) Conditional on rs3208305, rs3289, and rs117199990.

**Figure 4:**
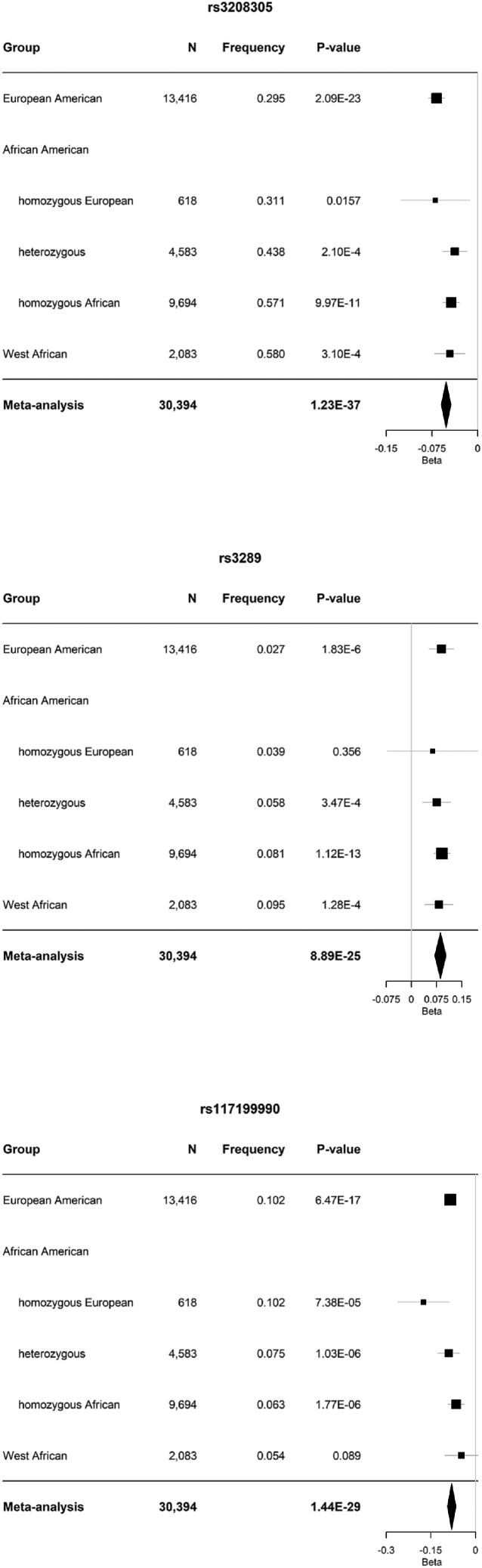
Forest plots depicting the results of the meta-analysis across European Americans, the three ancestral strata of African Americans, and West Africans “Frequency” indicates the frequency of the alternate allele. Error bars represent 95% confidence intervals.

We further tested the association of genotype on serum triglycerides at 1,623 variants in 2,083 West Africans and 916 variants in 13,416 unrelated European Americans and combined all results in meta-analysis. Of 1,878 total variants, associations at 302 were genome-wide significant (Table S6). The most strongly associated variant was rs3208305 (NC_000008.11:g.19966137A>T; $p=1.23\times 10^{-37}$; Figure 3A). Conditional on rs3208305, 78 associated variants remained genome-wide significant, and the most strongly associated variant was rs3289 (NC_000008.11:g.19965681T>C; $p=3.12\times 10^{-16}$; Figure 3B; Table S7). Conditional on rs3208305 and rs3289, 72 associated variants remained genome-wide significant, and the most strongly associated variant was rs117199990 (NC_000008.11:g.19963405C>T; $p=4.99\times 10^{-11}$; Figure 3C; Table S8). Conditional on rs3208305, rs3289, and rs117199990, no genome-wide significant associations remained (Figure 3D; Table S9). We then reanalyzed association for these three variants in a multivariable model. As expected, all three associations remained significant with no effect size heterogeneity but with reduced effect size estimates compared with single-marker analysis (Table 1; Figure 4). For all three variants, the absence of effect size heterogeneity is consistent with the effect of allelic substitution being independent of background. All three variants are noncoding and are expression quantitative trait loci (eQTLs) in blood, adipose tissue, and other tissues (Table S10).

**Table 1:** Multivariable analysis of conditionally independent associations

RSID	REF	ALT	β	SE	p	Direction^b^	HetChiSq	HetDf	HetPval	HetISq (%)	AFR^a^		EUR^a^	
											β	SE	β	SE
rs3208305	A	T	−0.0329	0.0045	1.44 × 10^−13^	–	7.610	4	0.1070	47.44	−0.0936	0.0046	−0.1112	0.0015
rs3289	T	C	0.0703	0.0086	4.44 × 10^−16^	+++++	0.305	4	0.9895	0.00	0.1654	0.0088	0.1554	0.0043
rs117199990	C	T	−0.0514	0.0078	4.57 × 10^−11^	–	7.681	4	0.1040	47.92	−0.1393	0.0090	−0.1797	0.0023

a Reproduced from Graham et al.^39^

b The five strata are European American, African American with homozygous African locus-specific ancestry, African American with heterozygous locus-specific ancestry, African American with homozygous European locus-specific ancestry, and West African.

At the most strongly associated variant, rs3208305, the frequency of the allele (*A*) associated with higher serum triglycerides was higher in the European background (68.9%) than in the African background (42.9%) among African Americans (Figure 4). Given the consistency of this pattern with the locus-specific ancestry effect, we estimated how much of the locus-specific ancestry effect was explained by rs3208305. Conditional on rs3208305, the locus-specific ancestry effect was insignificant (Z=1.14,p=0.254), indicating that rs3208305 by itself was sufficient to explain the locus-specific ancestry effect.

**Figure 5:**
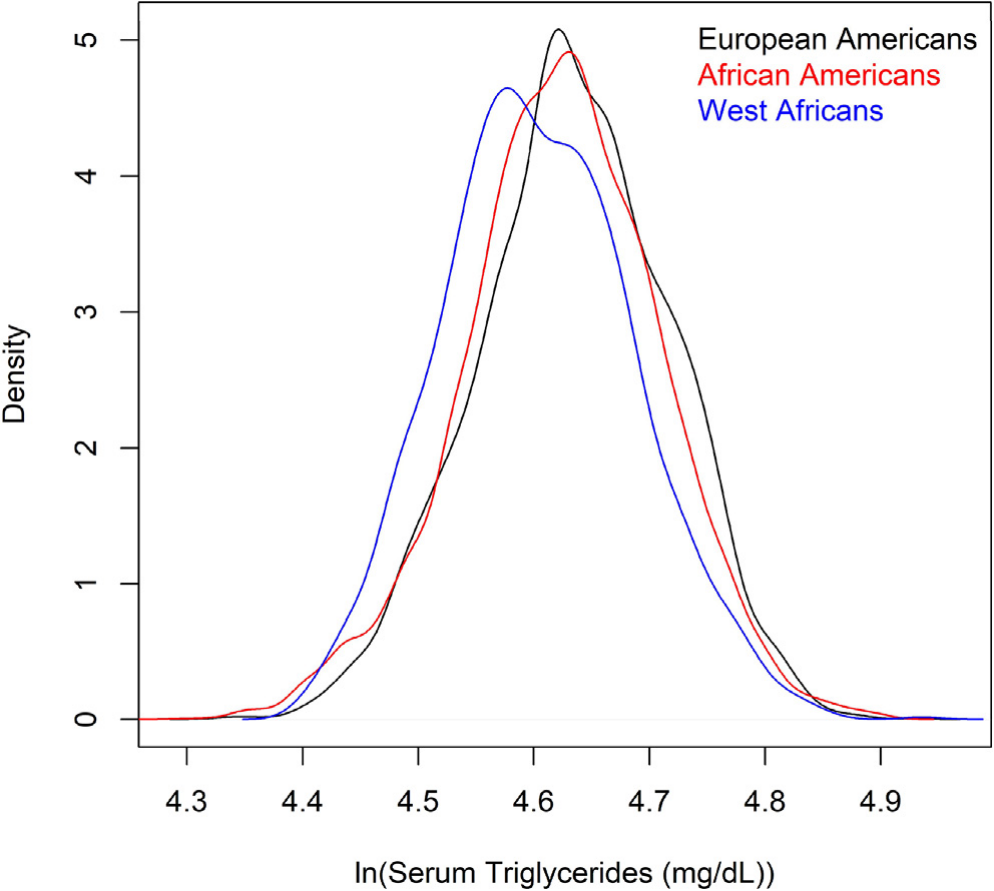
Predicted levels of serum triglycerides The genetic score accounted for the effects of age, sex, rs3208305, rs3289, and rs117199990.

In previous meta-analysis of 1,522,700 individuals from 316 studies, rs117199990 was identified as the most strongly associated variant in the 184.6 kb interval.^39^ To generate a baseline genetic score, we used the intercept and age and sex effects estimated from our meta-analysis of the multivariable model. Based on an independent set of 7,228 African Americans, 4,120 European Americans, and 1,386 West Africans, the baseline genetic score was positively correlated with observed serum triglycerides (r=0.2956). The genetic score including the effect for rs117199990 reported in the previous meta-analysis was less positively correlated with observed serum triglycerides (r=0.2420). In contrast, using the three conditionally independent variants and effect sizes we estimated, the genetic score was more strongly positively correlated with observed serum triglycerides (r=0.2959). Additionally, this genetic score predicted an interethnic difference, with European Americans predicted to have higher serum triglycerides than West Africans (Figure 5).

## Discussion

Most existing tests that integrate locus-specific ancestry and association effects were formulated with a composite alternative hypothesis of (1) a locus-specific ancestry effect but not an association effect, (2) an association effect but not a locus-specific ancestry effect, or (3) a locus-specific ancestry effect and an association effect. Consequently, these tests are joint tests of ancestry or association, not joint tests of ancestry and association. In contrast, we describe a joint test of ancestry and association with a composite alternative hypothesis of a locus-specific ancestry effect and an association effect. Our test is constructed as a product of locus-specific ancestry and association effects rather than as a sum. The use of a structured association test ensures that the association effect is conditionally independent of the locus-specific ancestry effect. Our test uses a third piece of information, the allele frequencies in the ancestries, to determine whether the sign of the association effect is consistent with the locus-specific ancestry effect. Consequently, our test can leverage the presence of a locus-specific ancestry effect to boost power to detect association while fine mapping the locus-specific ancestry effect by ruling out variants with combinations of allele frequencies and association effects inconsistent with the locus-specific ancestry effect. Joint tests based on sums of $\chi^{2}$ statistics can generate false positive results because of their failure to account for association effects that cannot causally explain locus-specific ancestry effects.

We observed a locus-specific ancestry effect at *LPL* such that an increasing amount of European ancestry correlated with higher serum triglycerides (or, equivalently, an increasing amount of African ancestry correlated with lower serum triglycerides). For rs3208305, Africans have more of the allele associated with lower triglycerides, a pattern consistent with the locus-specific ancestry effect. For rs3289 and rs117199990, Africans have more of the allele associated with higher serum triglycerides. Thus, these two associations are inconsistent with the locus-specific ancestry effect. Consequently, the joint test of ancestry and association detected rs3208305 but not rs3289 or rs117199990. Although the associations at rs3289 and rs1177199990 cannot be detected by the joint test, they can be detected using a standard test of association. All three variants have been reported previously to be associated with relevant phenotypes: rs3208305 with postprandial triglyceride levels,^40^ rs3289 with triglycerides,^41^^,^^42^ and rs117199990 with blood lipid metabolites.^43^ The previously reported index SNP rs328 (NP_000228.1:p.Ser474Ter), a nonsense variant, was associated with serum triglycerides in unconditional, single-marker analysis.^39^ However, after accounting for locus-specific ancestry effects, conditioning on rs3208305 and rs117199990 eliminated all of the association at rs328. Furthermore, genetic scores based on rs3208305, rs3289, and rs117199990 yielded improved prediction of serum triglycerides compared with using just rs117199990, including the prediction of an interethnic difference.

The interval of the locus-specific ancestry effect contains only one gene, *LPL*. We detected no associations at coding variants. The top index variant, rs3208305, tags rs13702 (NC_000008.11:g.19966981T>C), with strong linkage disequilibrium (LD) in both Africans ($r^{2}=0.929$) and European Americans ($r^{2}=0.997$). rs13702 is located in a binding site for miR-410 in the 3′ UTR and is associated with loss of binding.^44^^,^^45^ We hypothesize that loss of microRNA (miRNA) binding leads to escape from miRNA silencing, higher gene expression, and lower serum triglycerides. The second index variant, rs3289, is directly associated with the gain of a binding site for miR-145 in the 3′ UTR.^44^ In this case, we hypothesize that gain of miRNA binding leads to miRNA silencing, lower gene expression, and higher serum triglycerides. The third index variant, rs117199990, tags rs1803924 (NC_000008.11:g.19966163C>T), with strong LD in both Africans ($r^{2}=0.863$) and European Americans ($r^{2}=0.929$). rs1803924 is located in a binding site for miR-579 in the 3′ UTR and is associated with loss of binding.^44^^,^^45^ As with rs13702, we hypothesize that loss of miRNA binding leads to lower serum triglycerides.

In summary, we have derived a joint test of ancestry and association based on intersection-union testing, in contrast to existing joint tests of ancestry or association based on union-intersection testing. We illustrate the test by examining the association of *LPL* with serum triglycerides in 30,394 African Americans, European Americans, and West Africans. In admixed African Americans, increasing amounts of European ancestry correlated with increased serum triglycerides. Iterative conditional analysis revealed three associated variants, all annotated as eQTLs. The most strongly associated variant, rs3208305, showed allelic differentiation between European Americans and West Africans that was sufficient to explain the locus-specific ancestry effect. Controlling for the effects of locus-specific ancestry and multiple variants yielded effect size estimates with no heterogeneity. Collectively, our results underscore the fact that proper analytical methods are more important than millions of samples in order to unlock the power of genetic diversity. Given that populations and individuals of mixed ancestry are the rule, not the exception, our joint test of ancestry and association offers a powerful approach for the analysis of genetically diverse samples with proper control of locus-specific and genome-wide ancestry.

## Data and code availability

The datasets used for the analyses in this manuscript were obtained from dbGaP through dbGaP accession study numbers phs000280.v2.p1 (ARIC), phs000285.v3.p2 (CARDIA), phs000284.v1.p1 (CFS), phs000007.v32.p13 (FHS), phs001238.v2.p1 (GENOA), phs000286.v4.p1 (JHS), phs000209.v13.p3 (MESA), phs000433.v1.p1 (SIGNET), and phs000200.v12.p3 (WHI). The HUFS and AADM datasets are available from C.N.R. upon reasonable request.

## Web resources

Bcftools (v.1.9), http://www.htslib.org/download/
Bioconductor package GENESIS (v.2.24.0), https://bioconductor.org/packages/release/bioc/html/GENESIS.html
CheckVCF.py (v.1.4), https://github.com/zhanxw/checkVCF
GCTA (v.1.93.2beta), https://yanglab.westlake.edu.cn/software/gcta/#Download
Liftover, https://genome-store.ucsc.edu/
METAL (v.2018-08-28), https://github.com/statgen/METAL
OMIM: https://www.omim.org/
PLINK (v.1.90b2k), https://www.cog-genomics.org/plink/
R package RMediation (v.1.1.4), https://cran.r-project.org/web/packages/RMediation/index.html
RFMix (v.1.5.4), https://www.dropbox.com/s/cmq4saduh9gozi9/RFMix_v1.5.4.zip
Vcftools (v.0.1.16), https://github.com/vcftools/vcftools

## Supplemental information

Data S1. Tables S1 and S5–S9

Document S2. Article plus supplemental information
