# Supplementary material for: Universal genome-wide association studies: Powerful joint ancestry and association testing
Source: HGG Adv. 2023 Aug 30;4(4):100235. doi: 10.1016/j.xhgg.2023.100235 (PMC10507155; doi:10.1016/j.xhgg.2023.100235)
Supplement: Document S1. Figure S1 and Tables S2–S4 and S10 [file mmc1.pdf]

HGGA, Volume *4*

Supplemental information

Universal genome-wide association studies: Powerful joint ancestry and association testing

Daniel Shriner, Amy R. Bentley, Mateus H. Gouveia, Elisabeth F. Heuston, Ayo P. Doumatey, Guanjie Chen, Jie Zhou, Adebowale Adeyemo, and Charles N. Rotimi

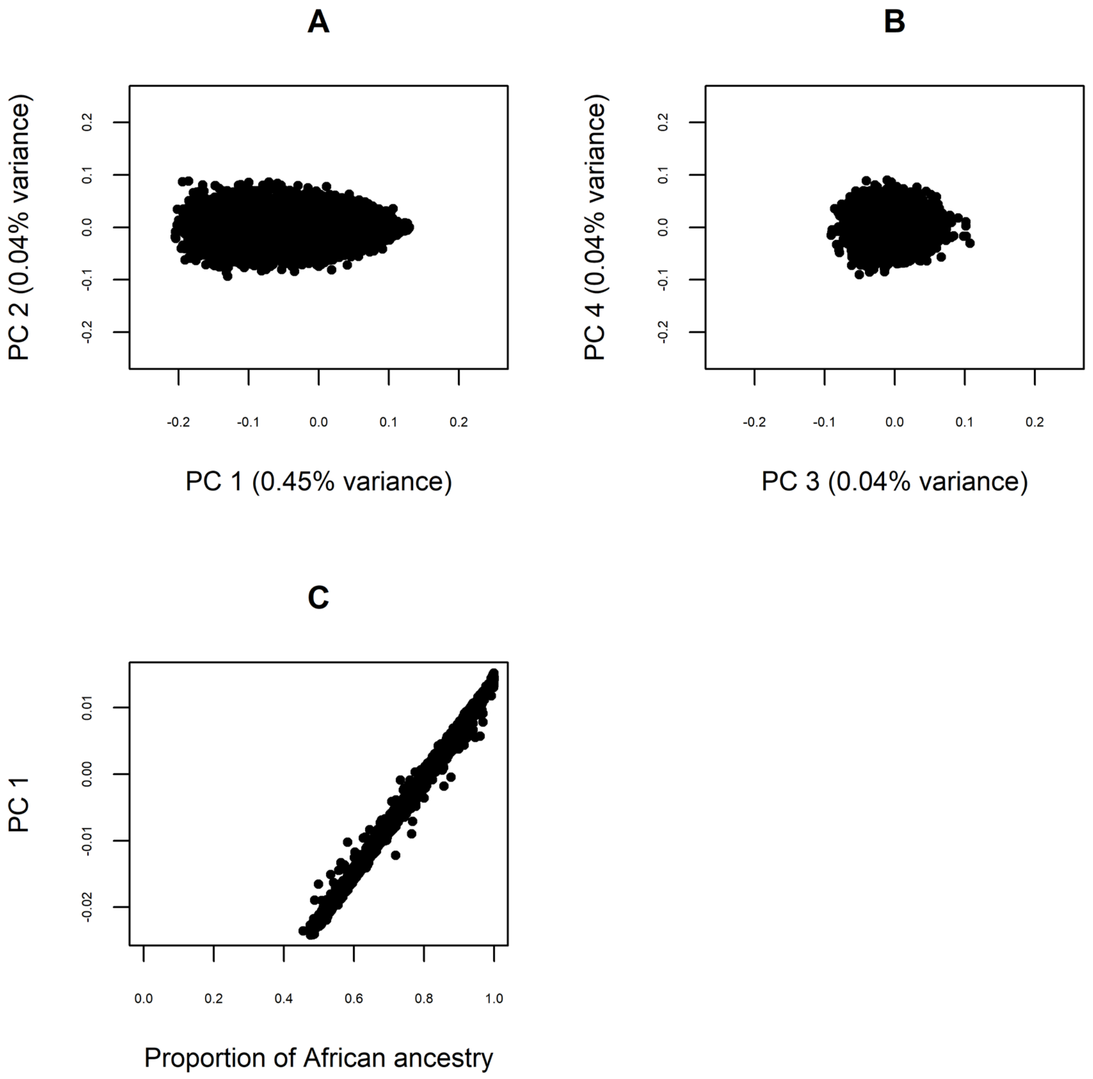

Figure S1. Principal components analysis of the African American data. A) Principal components (PC) 1 and 2. B) Principal components 3 and 4. C) Correlation of the proportion of African ancestry with principal component 1 ($r = 0.998$).

Table S2. Population names and abbreviations.

Population	Code	Metapopulation	Ancestry	Number available	Number included
African Caribbean in Barbados	ACB	AFR	African	96	34
People with African Ancestry in Southwest USA	ASW	AFR	African	61	1
Esan in Nigeria	ESN	AFR	African	99	93
Gambian in Western Division, Mandinka	GWD	AFR	African	113	87
Luhya in Webuye, Kenya	LWK	AFR	African	99	77
Mende in Sierra Leone	MSL	AFR	African	85	76
Yoruba in Ibadan, Nigeria	YRI	AFR	African	108	103
Colombians in Medellín, Colombia	CLM	AMR	Native American	94	0
People with Mexican Ancestry in Los Angeles, CA, USA	MXL	AMR	Native American	64	3
Peruvians in Lima, Peru	PEL	AMR	Native American	85	20
Puerto Ricans in Puerto Rico	PUR	AMR	Native American	104	0
Chinese Dai in Xishuangbanna, China	CDX	EAS	East Asian	93	38
Han Chinese in Beijing, China	CHB	EAS	East Asian	103	85
Southern Han Chinese	CHS	EAS	East Asian	105	97
Japanese in Tokyo, Japan	JPT	EAS	East Asian	104	62
Kinh in Ho Chi Minh City, Vietnam	KHV	EAS	East Asian	99	34
Utah residents (CEPH) with Northern and Western European ancestry	CEU	EUR	European	99	12
Finnish in Finland	FIN	EUR	European	99	31
British in England and Scotland	GBR	EUR	European	91	9
Iberian Populations in Spain	IBS	EUR	European	107	51
Toscani in Italia	TSI	EUR	European	107	33
Bengali in Bangladesh	BEB	SAS	South Asian	86	0
Gujarati Indians in Houston, TX, USA	GIH	SAS	South Asian	103	24
Indian Telugu in the UK	ITU	SAS	South Asian	102	21
Punjabi in Lahore, Pakistan	PJL	SAS	South Asian	96	4
Sri Lankan Tamil in the UK	STU	SAS	South Asian	102	18

Table S3. Reliability of locus-specific ancestry inference.

	Estimated Ancestry				
Reference Panel	African	Native American	East Asian	European	South Asian
AFR	98.96%	0.01%	0.03%	0.95%	0.05%
AMR	0.07%	97.77%	0.00%	2.16%	0.01%
EAS	0.00%	0.04%	99.56%	0.26%	0.13%
EUR	0.32%	0.12%	0.48%	99.06%	0.02%
SAS	0.00%	0.04%	0.14%	0.64%	99.18%

Table S4. Estimated genome-wide ancestral proportions among African Americans.

Study	African*	Native American	East Asian	European	South Asian
ARIC	0.820 [0.001, 1]	0.007 [0, 0.275]	0.007 [0, 0.032]	0.163 [0, 0.985]	0.003 [0, 0.086]
CFS	0.792 [0.288, 0.975]	0.007 [0, 0.246]	0.007 [0, 0.080]	0.191 [0.021, 0.703]	0.003 [0, 0.050]
GENOA	0.826 [0.358, 0.986]	0.007 [0, 0.168]	0.007 [0, 0.032]	0.157 [0.004, 0.618]	0.003 [0, 0.048]
HUFS	0.800 [0, 1]	0.007 [0, 0.449]	0.010 [0, 0.973]	0.180 [0, 0.949]	0.003 [0, 0.277]
JHS	0.822 [0, 0.986]	0.007 [0, 0.118]	0.007 [0, 0.992]	0.161 [0.001, 0.763]	0.003 [0, 0.044]
MESA	0.777 [0.223, 1]	0.007 [0, 0.299]	0.007 [0, 0.501]	0.206 [0, 0.735]	0.003 [0, 0.242]
SIGNET	0.873 [0.001, 1]	0.008 [0, 0.114]	0.004 [0, 0.083]	0.113 [0, 0.992]	0.002 [0, 0.214]
WHI	0.767 [0, 0.999]	0.007 [0, 0.467]	0.007 [0, 0.992]	0.216 [0, 0.994]	0.003 [0, 0.445]

* Mean [minimum, maximum]

Table S10: Expression quantitative trait loci from bulk tissue.

Gencode ID	Gene Symbol	Variant ID	RSID	*P* -Value	Normalized Effect Size	Tissue
ENSG00000175445.14	*LPL*	chr8_19966137_A_T_b38	rs3208305	5.18E-17	0.21	Nerve - Tibial
ENSG00000175445.14	*LPL*	chr8_19966137_A_T_b38	rs3208305	1.31E-07	0.09	Adipose - Subcutaneous
ENSG00000175445.14	*LPL*	chr8_19966137_A_T_b38	rs3208305	3.08E-07	0.22	Whole Blood
ENSG00000175445.14	*LPL*	chr8_19966137_A_T_b38	rs3208305	8.59E-07	0.23	Testis
ENSG00000175445.14	*LPL*	chr8_19966137_A_T_b38	rs3208305	8.94E-07	0.21	Thyroid
ENSG00000175445.14	*LPL*	chr8_19965681_T_C_b38	rs3289	2.32E-12	-0.29	fat
ENSG00000175445.14	*LPL*	chr8_19963405_C_T_b38	rs117199990	1.78E-15	0.56	Whole Blood
ENSG00000175445.14	*LPL*	chr8_19963405_C_T_b38	rs117199990	3.54E-11	2.15	monocyte_CD16_naive
ENSG00000175445.14	*LPL*	chr8_19963405_C_T_b38	rs117199990	7.12E-11	0.86	blood
ENSG00000175445.14	*LPL*	chr8_19963405_C_T_b38	rs117199990	5.02E-08	0.23	Nerve - Tibial
ENSG00000175445.14	*LPL*	chr8_19963405_C_T_b38	rs117199990	2.32E-07	0.74	Spleen

Acknowledgements

The Atherosclerosis Risk in Communities study has been funded in whole or in part with Federal funds from the National Heart, Lung, and Blood Institute, National Institutes of Health, Department of Health and Human Services, under contract numbers HHSN268201700001I, HHSN268201700002I, HHSN268201700003I, HHSN268201700004I, and HHSN268201700005I. The authors thank the staff and participants of the ARIC study for their important contributions. Funding for the GENEVA substudy was provided by National Human Genome Research Institute grant U01HG004402 (E. Boerwinkle). Support for the Coronary Artery Risk Development in Young Adults study was provided by NHLBI grant numbers HHSN268201300025C, HHSN268201300026C, HHSN268201300027C, HHSN268201300028C, and HHSN268201300029C (C. E. Lewis, D. Lloyd-Jones, P. Schreiner, S. Sidney, and J. Shikany). Support for the Cleveland Family Study was provided by NHLBI grant numbers R01 HL46380 and R01 HL113338. The Framingham Heart Study is conducted and supported by the National Heart, Lung, and Blood Institute (NHLBI) in collaboration with Boston University (contracts N01-HC-25195, HHSN268201500001I, and 75N92019D00031). This manuscript was not prepared in collaboration with investigators of the Framingham Heart Study and does not necessarily reflect the opinions or views of the Framingham Heart Study, Boston University, or NHLBI. Funding to support the Omni cohort recruitment, retention, and examination was provided by NHLBI contracts N01-HC-25195, HHSN268201500001I, and 75N92019D00031, as well as NHLBI grants R01-HL070100, R01-HL076784, R01-HL49869, and U01-HL-053941. SHARe Illumina genotyping was provided under an agreement between Illumina and Boston University. Support for GENOA was provided by the National Heart, Lung and Blood Institute (HL054457, HL054464, HL054481, HL119443, and HL087660) of the

National Institutes of Health. We would like the thank the Mayo Clinic Genotyping Core, the DNA Sequencing and Gene Analysis Center at the University of Washington, and the Broad Institute for their genotyping and sequencing services. We would like to thank the GENOA participants. This manuscript was not prepared in collaboration with investigators from the Genetic Epidemiology Network of Arteriopathy and does not necessarily reflect the opinions or views of the Genetic Epidemiology Network of Arteriopathy or NHLBI. The Jackson Heart Study (JHS) is supported and conducted in collaboration with Jackson State University (HHSN268201800013I), Tougaloo College (HHSN268201800014I), the Mississippi State Department of Health (HHSN268201800015I/HHSN26800001) and the University of Mississippi Medical Center (HHSN268201800010I, HHSN268201800011I and HHSN268201800012I) contracts from the National Heart, Lung, and Blood Institute (NHLBI) and the National Institute for Minority Health and Health Disparities (NIMHD). The authors wish to thank the staffs and participants of the JHS. Funding for CARe genotyping was provided by NHLBI Contract N01-HC-65226. MESA and the MESA SHARe project are conducted and supported by the National Heart, Lung, and Blood Institute (NHLBI) in collaboration with MESA investigators. Support for MESA is provided by contracts N01-HC95159, N01-HC-95160, N01-HC-95161, N01-HC-95162, N01-HC-95163, N01-HC-95164, N01-HC-95165, N01-HC95166, N01-HC-95167, N01-HC-95168, N01-HC-95169, UL1-RR-025005, and UL1-TR-000040. Funding for SHARe genotyping was provided by NHLBI Contract N02-HL-64278. Genotyping was performed at Affymetrix (Santa Clara, California, USA) and the Broad Institute of Harvard and MIT (Boston, Massachusetts, USA) using the Affymetrix Genome-Wide Human SNP Array 6.0. This manuscript was not prepared in collaboration with MESA investigators and does not necessarily reflect the opinions or views of MESA, or the NHLBI. Support for SIGNET

is provided by R01-DK084350 (M. M. Sale), U01-NS041588 (G. Howard), M01-RR001070 (J. K. Fernandes), P20-RR017696 (K. L. Kirkwood), and P60-AR049459 (G. S. Gilkeson) and by the W. M. Keck Foundation (W. T. Garvey). The WHI program is funded by the National Heart, Lung, and Blood Institute, National Institutes of Health, U.S. Department of Health and Human Services through contracts HHSN268201600018C, HHSN268201600001C, HHSN268201600002C, HHSN268201600003C, and HHSN268201600004C. This manuscript was not prepared in collaboration with investigators of the WHI, has not been reviewed and/or approved by the Women's Health Initiative (WHI), and does not necessarily reflect the opinions of the WHI investigators or the NHLBI. Funding for WHI SHARe genotyping was provided by NHLBI contract N02-HL-64278.
