# Supplementary material for: Universal genome-wide association studies: Powerful joint ancestry and association testing
Source: HGG Adv. 2023 Aug 30;4(4):100235. doi: 10.1016/j.xhgg.2023.100235 (PMC10507155; doi:10.1016/j.xhgg.2023.100235)
Supplement: Document S2. Article plus supplemental information [file mmc3.pdf]

HGG
Advances

ARTICLE

Universal genome-wide association studies: Powerful joint ancestry and association testing

Daniel Shriner,[1] Amy R. Bentley,[1] Mateus H. Gouveia,[1] Elisabeth F. Heuston,[1] Ayo P. Doumatey,[1] Guanjie Chen,[1] Jie Zhou,[1] Adebowale Adeyemo,[1] and Charles N. Rotimi[1,2,*]

Summary

The vast majority of human populations and individuals have mixed ancestry. Consequently, adjustment for locus-specific ancestry is essential for genetic association studies. To empower association studies for all populations, it is necessary to integrate effects of locus-specific ancestry and genotype. We developed a joint test of ancestry and association that can be performed with summary statistics, is independent of study design, can take advantage of locus-specific ancestry effects to boost power in association testing, and can utilize association effects to fine map admixture peaks. We illustrate the test using the association between serum triglycerides and *LPL*. By combining data from African Americans, European Americans, and West Africans, we identify three conditionally independent variants with varying amounts of ancestrally differentiated allele frequencies. Using out-of-sample data, we demonstrate improved prediction achievable by accounting for multiple causal variants and locus-specific ancestry effects at a single locus.

Introduction

The process of admixture refers to interbreeding between previously isolated populations and results in individuals with mixed ancestry. The idea of admixture mapping as a gene mapping tool was described in 1954[1] but did not become practical at the genome-wide scale until the early 2000s with the accumulation of marker data[2] and the development of methods.[3] The use of mixed ancestry to map trait genes across the genome was first possible for African Americans,[2,3] although it is now recognized that the overwhelming majority of human populations and individuals have mixed ancestry.[4–6] In a chromosomal region containing an allele that increases risk or a trait value, there should be an excess of ancestry from whichever parental population has a higher frequency of the risk or trait-increasing allele at that locus.[3] Admixture mapping is based on correlating allele frequency differences between parental populations with the phenotype, which is the same information that determines whether population stratification is a confounder in genetic association studies.

A union-intersection test (UIT) involves the composite null hypothesis that all null hypotheses are true against the composite alternative hypothesis that at least one alternative hypothesis is true.[7,8] In the gene mapping context, the composite alternative hypothesis is that (1) there is a locus-specific ancestry effect but no association effect, (2) there is an association effect but no locus-specific ancestry effect, or (3) there are both locus-specific ancestry and association effects (Figure 1). The alternative hypothesis of a locus-specific ancestry effect but no association effect is tantamount to linkage without association and is typically not of interest. The alternative hypothesis of an association effect but no locus-specific ancestry effect is more powerfully tested using a standard association test. UITs are readily constructed by taking the sums of test statistics. Several UITs that are joint ancestry or association tests have been described for family data[9] or population data.[10–13] This class of tests includes the SUM test[10] and the test implemented in Tractor.[13] Tests that are based on χ^2 statistics ignore the information contained in the signs of the association and locus-specific ancestry effects. Consequently, a test based on χ^2 statistics could yield a false positive result if there is an association between increased risk or a trait value and an excess of ancestry from whichever parental population has a lower frequency of the risk or trait-increasing allele at that locus.

An intersection-union test (IUT) involves a composite null hypothesis that at least one null hypothesis is true against the composite alternative hypothesis that all alternative hypotheses are true.[7,8] In the gene mapping context, the composite alternative hypothesis is that there are both locus-specific ancestry and association effects (Figure 1). A well-known example of an IUT in gene mapping is the transmission-disequilibrium test, which is a joint test of linkage and association and is based on the product of linkage and linkage disequilibrium.[14] A second example is the MIX test,[10] which is a product of locus-specific ancestry and association effects but is defined solely for the case-only design.[3,15,16] The case-only design

[1]Center for Research on Genomics and Global Health, National Human Genome Research Institute, Bethesda, MD 20892, USA
[2]Lead contact
*Correspondence: rotimic@mail.nih.gov
https://doi.org/10.1016/j.xhgg.2023.100235.
This is an open access article under the CC BY license (http://creativecommons.org/licenses/by/4.0/).

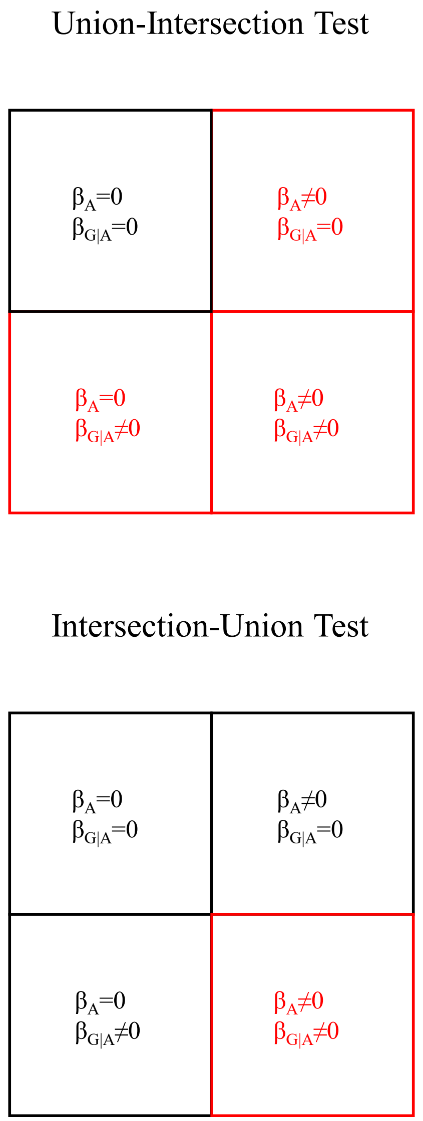

Figure 1. Contrasting design of the union-intersection and intersection-union tests
β_A denotes the effect of locus-specific ancestry, and $\beta_{G|A}$ denotes the effects of genotype conditional on locus-specific ancestry. Black represents composite null hypotheses, and red represents composite alternative hypotheses.

is not robust to misspecification of parental allele frequencies, and controls are necessary to address the possibility that cases and controls might have different ancestry proportions on average. A third example is the BMIX test,[17] which suffers from the same loss of information as other tests based on χ^2 statistics.

Here, we derive an IUT that is a joint ancestry and association test. This test is applicable for any study design and phenotype that can be analyzed using a generalized linear mixed model. Furthermore, this test is based on signed z statistics rather than χ^2 statistics and so retains validity across all configurations of locus-specific ancestry and genotype states. We illustrate the utility of this test by untangling locus-specific ancestry and association effects at *LPL* (MIM: 609708) for serum triglycerides.[18,19]

Materials and methods

IUT

At a marker, let z_A represent the Z score for a locus-specific ancestry effect and let $z_{G|A}$ represent the Z score for an association effect of genotype conditional on locus-specific ancestry. The product of these two Z scores is the test statistic for the joint test of ancestry and association. The product can only be nonzero if both Z scores are nonzero. The product follows the product normal distribution. We used the R package RMediation[20] to evaluate p values and quantiles from the product normal distribution.

The signs of the Z scores convey useful information that would be lost in a test based on χ^2 statistics. To illustrate, consider two-way admixed individuals. Assuming no parent-of-origin effects, there are three strata based on locus-specific ancestry, two homozygous and one heterozygous. Let locus-specific ancestry be coded as a categorical variable based on the number of alleles inherited from a specified parental population: 0 (homozygous for one ancestry), 1 (heterozygous), or 2 (homozygous for the other ancestry). Let genotype be coded as 0, 1, or 2 copies of the effect allele. Suppose that the locus-specific ancestry effect is positive. Then, that locus-specific ancestry effect can be explained by a positive association effect if the frequency of the effect allele is positively correlated with locus-specific ancestry or by a negative association effect if the frequency of the effect allele is negatively correlated with locus-specific ancestry. Under the assumption that the effect of allelic substitution is independent of background, a positive product constitutes evidence for joint ancestry and association, whereas a negative product does not. For this reason, the product test is one tailed. To operationalize this logic, let f_0 be the frequency of the effect allele in the stratum of locus-specific ancestry coded 0 and let f_2 be the frequency of the effect allele in the stratum of locus-specific ancestry coded 2. The test statistic is defined as $\mathrm{sign}(f_2 - f_0)Z_A Z_{G|A}$. This test is designed for pairwise comparisons assuming at least two ancestral strata.

Population descriptors

The definition of admixture as the process of intermating between previously isolated populations involves, at a minimum, three populations (an admixed population and two parental or source populations) and two timescales. In our study, the descriptor African American refers to descendants of the Middle Passage. Admixed African Americans resulted from intermating over the last few hundred years. The descriptors African and European refer to the two primary source populations. These two descriptors are intentionally broad and inclusive in order to reflect the genetic diversity in both sources. The timescale of isolation encompasses the tens to hundreds of thousands of years since the split of anatomically modern humans into lineages that predated human dispersal out of Africa.

Studies

We integrated individual-level data from eight genetic epidemiology studies comprising 23,643 African Americans (Table S1): the Atherosclerosis Risk in Communities (ARIC) study,[21] the Cleveland Family Study (CFS),[22] the Genetic Epidemiology Network of Arteriopathy (GENOA),[23] the Howard University Family Study (HUFS),[24] the Jackson Heart Study (JHS),[25] the Multi-Ethnic Study of Atherosclerosis (MESA),[26] the Sea Islands Genetic Network (SIGNET),[27] and the Women's Health Initiative (WHI).[28] We integrated individual-level data from five genetic

epidemiology studies comprising 17,684 European Americans (Table S1): ARIC,[21] the Coronary Artery Risk Development in Young Adults (CARDIA) study,[29] the Framingham Heart Study (FHS),[30] GENOA,[23] and MESA.[26] We also analyzed individual-level data for 3,583 West Africans from Ghana or Nigeria (Table S1) from the Africa America Diabetes Mellitus (AADM) study.[31] Ethical approval was obtained from the National Institutes of Health and from the ethical committees in each study site. All participants gave written informed consent.

Phenotype and covariates

Serum triglyceride (mg/dL) measurements for HUFS and AADM were made on fasting samples and determined enzymatically, as previously reported.[19,31] For those studies accessed through dbGaP, data were extracted from pht000114 (ARIC), pht001588 (CARDIA), pht001902 (CFS), pht006027 (FHS), pht006655 (GENOA), pht008729 (JHS), pht001116 (MESA), pht002436 (SIGNET), and pht003419 (WHI). Data for serum triglycerides were cleaned based on inspection of the distribution within each study separately to remove outliers and then natural-log transformed. Age was extracted for the visit corresponding to the serum triglyceride measurement.

Genotype cleaning and imputation

For each study, we followed the same genotype cleaning and imputation steps. First, markers that were strand ambiguous or monomorphic were removed. Using PLINK, we then filtered for individual-level missingness of 0.1, per-marker missingness of 0.05, and a Hardy-Weinberg equilibrium p value of 1×10^{-10}. We used the liftover tool to migrate coordinates from NCBI/B36 to GRCh37. We then used the bcftools plugin fixref to orient all alleles as reference and alternate according to the file human_g1k_v37.fasta. Next, we used the Python script checkVCF.py to clean the datasets prior to imputation with the TOPMed server and the r2 reference panel (coordinates based on GRCh38). Imputed data were filtered based on an rsq quality score of 0.3 and a minor allele frequency of 0.5%. Imputed datasets were merged using bcftools.

Inference of locus-specific ancestry

For each of the eight studies of African Americans, we inferred locus-specific ancestry using RFMix.[32] We constructed a five-way reference panel based on a subset of 2,504 individuals in phase 3 of the 1000 Genomes Project[5] and individual admixture proportions.[6] Population identifiers as defined by the 1000 Genomes Project[5] are reproduced in Table S2. From the AFR metapopulation, we retained 471 individuals (34 ACB, 1 ASW, 93 ESN, 87 GWD, 77 LWK, 76 MSL, and 103 YRI) with predominantly Central African, Eastern African, Omotic, Southern African, West-Central African, or Western African ancestry. From the AMR metapopulation, we retained 23 individuals (20 PEL and 3 MXL) with predominantly Amerindian or Circumpolar ancestry. From the EAS metapopulation, we retained 316 individuals (38 CDX, 85 CHB, 97 CHS, 62 JPT, and 34 KHV) with predominantly Japanese, Sino-Tibetan, or Southeastern Asian ancestry. From the EUR metapopulation, we retained 136 individuals (51 IBS, 31 FIN, 12 CEU, 9 GBR, and 33 TSI) with predominantly Arabian, Northern African, Northern European, Southern European, or Western Asian ancestry. From the SAS metapopulation, we retained 67 individuals (24 GIH, 21 ITU, 4 PJL, and 18 STU) with predominantly South Indian or Southern Asian ancestry. We ran RFMix with the settings -n 5 and -w 0.1. By inferring locus-specific ancestry within the reference panel, instead of treating the reference panel as fixed, we estimated 99.15% reliability of assignment of locus-specific ancestry (Table S3). We inferred locus-specific ancestry for each of the eight studies separately (Table S4) and then merged the calls across the intersection of markers that were genotyped in all eight studies. Calls were recoded as 0, 1, or 2 alleles inherited from the African parental population at the locus.

Population structure

Principal-component analysis of the African data revealed that the top principal component separated Ghanaians from Nigerians.[33] Principal-component analysis of the European data revealed that the top principal component reflected a north-to-south cline.[34] Principal-component analysis of the African American data revealed that the first principal component correlated with the individual proportion of African ancestry (Figure S1). None of the top principal components correlated with study or genotyping array.

Admixture mapping

To test the effect of locus-specific ancestry on phenotype, we regressed the phenotype on locus-specific ancestry using a linear mixed model, adjusted for age, sex, and study as fixed effects and the relatedness matrix estimated from locus-specific ancestry as a random effect (which simultaneously controls genome-wide ancestry, known relatedness, and cryptic relatedness). To reduce confounding by shared environmental effects among close relatives, unrelated individuals were extracted using –grm-cutoff 0.046875 (i.e., between 4th and 5th degree relatives) in GCTA.[35] To perform the regression, we used the –mlma option in GCTA, which leaves out the chromosome being tested from the relatedness matrix. Note that this test does not require the phenotype to be stratified across ancestries or for there to be a significant effect of genome-wide ancestry.

Structured association testing

To test the effect of genotype on phenotype, we regressed the phenotype on imputed dosages using a linear mixed model, adjusted for age, sex, and study as fixed effects and the genetic relatedness matrix as a random effect. Unrelated individuals were extracted using –grm-cutoff 0.046875 in GCTA. We used the Bioconductor package GENESIS, leaving out the chromosome being tested from the genetic relatedness matrix. To account for admixture in African Americans, we stratified the association using three strata of locus-specific ancestry (i.e., homozygous African, heterozygous, and homozygous European), an approach robust to the unknown genetic mode of inheritance.[36,37] We combined the three sets of results across strata using the standard error scheme in METAL. Given that the genome-wide testing burden for association testing exceeds the genome-wide testing burden for admixture mapping in admixed African Americans by three orders of magnitude,[17] the genome-wide significance level was set at 5×10^{-8}. Annotation was retrieved from Ensembl release 105.[38]

Genetic score and prediction

We generated a genetic score accounting for the effects of age, sex, and genetic variants. For each genetic variant, we weighted the allelic dosage by the estimated allelic effect size. We used the nonoverlapping set of individuals excluded upon filtering for relatedness.

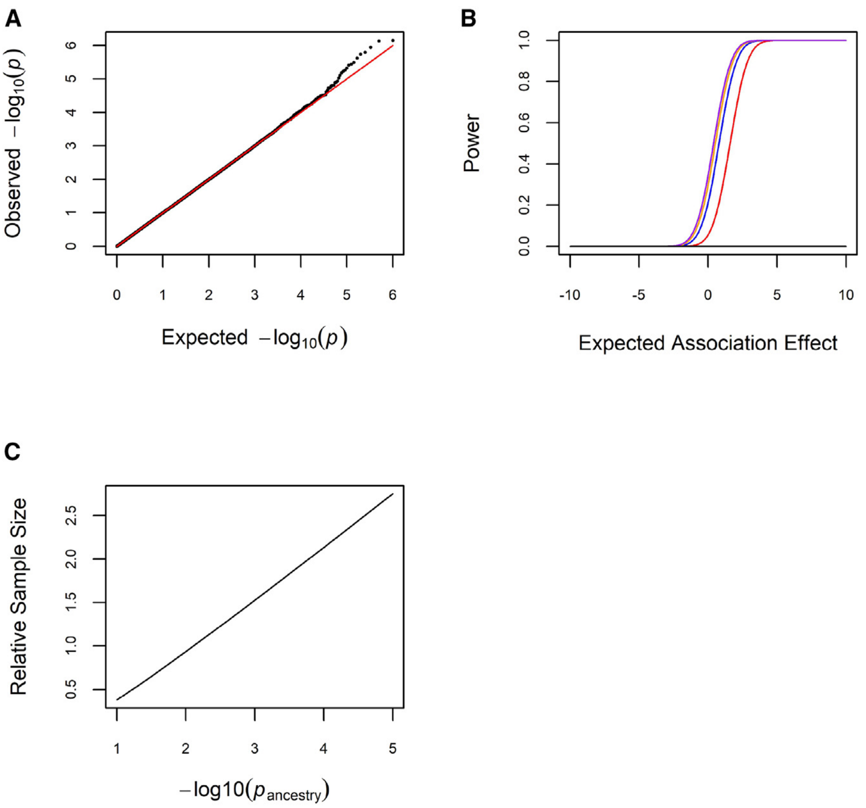

Figure 2. Performance of the joint ancestry and association test
(A) Type I error rate. The black line represents data simulated under the null hypothesis, and the red line represents the expected uniform distribution of p values under the null hypothesis.
(B) Power. We simulated the expected association effect as the mean of a normal distribution with unit variance and a realized locus-specific ancestry effect as a standardized Z score (black represents $Z_A = 0$, red represents $Z_A = 1$, blue represents $Z_A = 2$, orange represents $Z_A = 3$, and purple represents $Z_A = 4$). The locus-specific ancestry and association effects are statistically independent.
(C) Benchmarking. The x axis indicates $-\log_{10}(p_{ancestry})$, and the y axis indicates the sample size of the association test required to achieve genome-wide significance by the standard association test relative to the joint test.

Results

To integrate locus-specific ancestry and association effects, we derived an IUT to specifically test the composite alternative hypothesis of both locus-specific ancestry and association effects. Let Z_A represent the Z score for a locus-specific ancestry effect and let $Z_{G|A}$ represent the Z score for an association effect of genotype conditional on locus-specific ancestry. The product of these two Z scores is a test statistic for the joint test of ancestry and association. Under the null hypothesis, the test statistic is distributed as the product of two standard normal random variables. Conditioning association testing on locus-specific ancestry leads to the property that the two random variables are conditionally independent. By incorporating ancestral allele frequencies into the test, we constrain the test to be one tailed. To establish the validity of this test, we generated 1,000,000 statistical replicates of three independent random variates from the standard normal distribution and calculated the test statistic by multiplying the sign of the first random variate by the product of the other two random variates. For a significance level $\alpha = 0.05$, the type I error rate was 0.049779, indicating that the one-tailed product test was valid (Figure 2A). On an HP EliteBook 830 G8 Notebook with a single Intel i7 processor running at 3 GHz, runtime for 1,000,000 tests was 112 s. As expected, for a given locus-specific ancestry effect, power increased as the association effect increased (Figure 2B). Also as expected, for a given association effect, power increased as the locus-specific ancestry effect increased (Figure 2B).

We next benchmarked the joint ancestry and association test relative to the standard association test (Figure 2C). We performed this benchmarking analytically (i.e., without simulations) by working directly with Z statistics and p values. Specifically, $p_{association} = 5\times10^{-8}$ corresponds to $Z_{G|A} = 5.45$ and $p_{joint} = 5\times10^{-8}$ corresponds to $Z_A Z_{G|A} = 14.52$. To achieve $p_{joint} = 5\times10^{-8}$ given a genome-wide significant admixture mapping signal with $p_{ancestry} = 1\times10^{-5}$ ($Z_A = 4.42$), we require $Z_{G|A} = 3.29$, corresponding to $p_{association} = 1.01\times10^{-3}$ and reflecting an increase in power equivalent to a 2.75-fold larger sample size to detect association relative to the standard association test. To achieve $p_{joint} = 5\times10^{-8}$ given a genome-wide association signal with $p_{association} = 5\times10^{-8}$, we require $p_{ancestry} = 7.74\times10^{-3}$, indicating a loss of power to detect association relative to the standard association test if the locus-specific ancestry effect is small.

Given prior evidence for a locus-specific ancestry effect at *LPL* for serum triglycerides,[19] we estimated the size of this effect in 14,895 unrelated African Americans. Admixture mapping revealed an interval of 184.6 kb (from rs7821631 [NC_000008.11:g.19873864T>C] to rs13263007 [NC_000008.11:g.20058493C>T]), at which an increasing amount of European ancestry was associated with increased serum triglycerides ($Z = 2.76$, $p = 5.83\times10^{-3}$). *LPL* is the only gene in this interval.

We next tested the association of genotype on serum triglycerides at all 1,679 variants in the 184.6 kb interval in 14,895 unrelated African Americans (Table S5). A total of 103 variants were genome-wide significant. When we required directional consistency with the locus-specific ancestry effect, 49 variants were genome-wide significant,

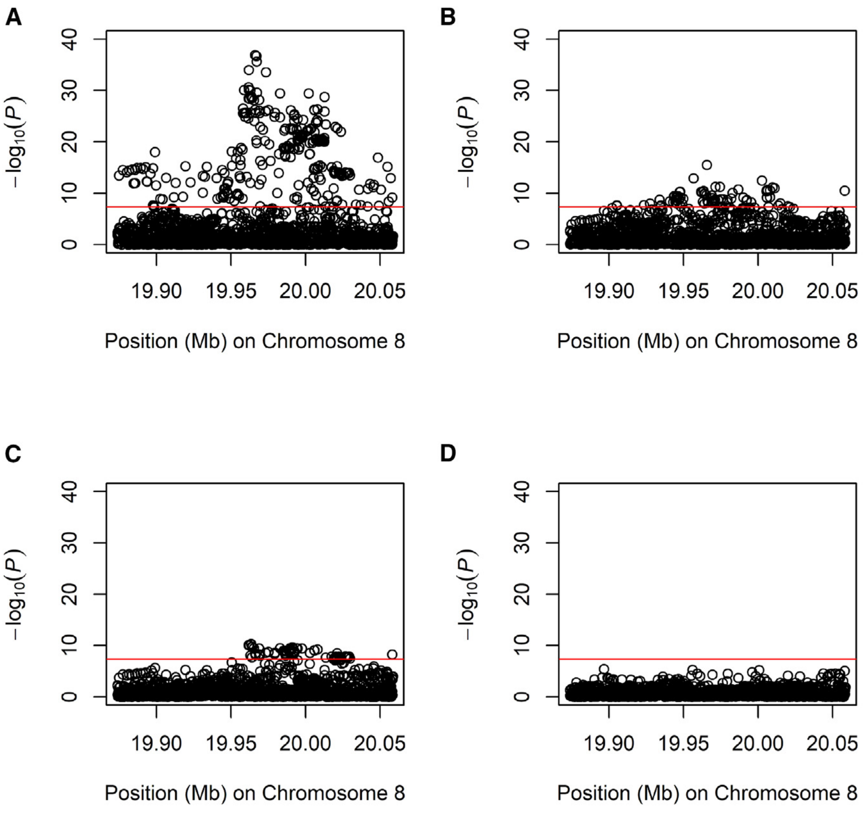

Figure 3. Meta-analysis of association results for Africans, African Americans, and Europeans
(A) Unconditional analysis.
(B) Conditional on rs3208305.
(C) Conditional on rs3208305 and rs3289.
(D) Conditional on rs3208305, rs3289, and rs117199990.

reflecting fine mapping of the locus-specific ancestry effect. The joint test yielded an increase of genome-wide significant variants from 49 to 66. All 17 additional variants had association p values below genome-wide significance (p values ranged from 1.39×10^{-7} to 6.17×10^{-8}). The increase in the number of variants genome-wide significant by the joint test compared with the standard association test reflects a gain in power.

We further tested the association of genotype on serum triglycerides at 1,623 variants in 2,083 West Africans and 916 variants in 13,416 unrelated European Americans and combined all results in meta-analysis. Of 1,878 total variants, associations at 302 were genome-wide significant (Table S6). The most strongly associated variant was rs3208305 (NC_000008.11:g.19966137A>T; $p = 1.23\times10^{-37}$; Figure 3A). Conditional on rs3208305, 78 associated variants remained genome-wide significant, and the most strongly associated variant was rs3289 (NC_000008.11:g.19965681T>C; $p = 3.12\times10^{-16}$; Figure 3B; Table S7). Conditional on rs3208305 and rs3289, 72 associated variants remained genome-wide significant, and the most strongly associated variant was rs117199990 (NC_000008.11:g.19963405C>T; $p = 4.99\times10^{-11}$; Figure 3C; Table S8). Conditional on rs3208305, rs3289, and rs117199990, no genome-wide significant associations remained (Figure 3D; Table S9). We then reanalyzed association for these three variants in a multivariable model. As expected, all three associations remained significant with no effect size heterogeneity but with reduced effect size estimates compared with single-marker analysis (Table 1; Figure 4). For all three variants, the absence of effect size heterogeneity is consistent with the effect of allelic substitution being independent of background. All three variants are noncoding and are expression quantitative trait loci (eQTLs) in blood, adipose tissue, and other tissues (Table S10).

At the most strongly associated variant, rs3208305, the frequency of the allele (*A*) associated with higher serum triglycerides was higher in the European background (68.9%) than in the African background (42.9%) among African Americans (Figure 4). Given the consistency of this pattern with the locus-specific ancestry effect, we estimated how much of the locus-specific ancestry effect was explained by rs3208305. Conditional on rs3208305, the locus-specific ancestry effect was insignificant ($Z = 1.14, p = 0.254$), indicating that rs3208305 by itself was sufficient to explain the locus-specific ancestry effect.

In previous meta-analysis of 1,522,700 individuals from 316 studies, rs117199990 was identified as the most strongly associated variant in the 184.6 kb interval.[39] To generate a baseline genetic score, we used the intercept and age and sex effects estimated from our meta-analysis of the multivariable model. Based on an independent set of 7,228 African Americans, 4,120 European Americans, and 1,386 West Africans, the baseline genetic score was positively correlated with observed serum triglycerides ($r = 0.2956$). The genetic score including the effect for rs117199990 reported in the previous meta-analysis was less positively correlated with observed serum triglycerides ($r = 0.2420$). In contrast, using the three conditionally independent variants and effect sizes we estimated, the genetic score was more strongly positively correlated with observed serum triglycerides ($r = 0.2959$). Additionally, this genetic score predicted an interethnic difference, with European Americans predicted to have higher serum triglycerides than West Africans (Figure 5).

Discussion

Most existing tests that integrate locus-specific ancestry and association effects were formulated with a composite

Table 1. **Multivariable analysis of conditionally independent associations**

											AFR[a]		EUR[a]	
RSID	REF	ALT	β	SE	p	Direction[b]	HetChiSq	HetDf	HetPval	HetISq (%)	β	SE	β	SE
rs3208305	A	T	−0.0329	0.0045	1.44×10^{-13}	–	7.610	4	0.1070	47.44	−0.0936	0.0046	−0.1112	0.0015
rs3289	T	C	0.0703	0.0086	4.44×10^{-16}	+++++	0.305	4	0.9895	0.00	0.1654	0.0088	0.1554	0.0043
rs117199990	C	T	−0.0514	0.0078	4.57×10^{-11}	–	7.681	4	0.1040	47.92	−0.1393	0.0090	−0.1797	0.0023

[a]Reproduced from Graham et al.[39]
[b]The five strata are European American, African American with homozygous African locus-specific ancestry, African American with heterozygous locus-specific ancestry, African American with homozygous European locus-specific ancestry, and West African.

alternative hypothesis of (1) a locus-specific ancestry effect but not an association effect, (2) an association effect but not a locus-specific ancestry effect, or (3) a locus-specific ancestry effect and an association effect. Consequently, these tests are joint tests of ancestry or association, not joint tests of ancestry and association. In contrast, we describe a joint test of ancestry and association with a composite alternative hypothesis of a locus-specific ancestry effect and an association effect. Our test is constructed as a product of locus-specific ancestry and association effects rather than as a sum. The use of a structured association test ensures that the association effect is conditionally independent of the locus-specific ancestry effect. Our test uses a third piece of information, the allele frequencies in the ancestries, to determine whether the sign of the association effect is consistent with the locus-specific ancestry effect. Consequently, our test can leverage the presence of a locus-specific ancestry effect to boost power to detect association while fine mapping the locus-specific ancestry effect by ruling out variants with combinations of allele frequencies and association effects inconsistent with the locus-specific ancestry effect. Joint tests based on sums of χ^2 statistics can generate false positive results because of their failure to account for association effects that cannot causally explain locus-specific ancestry effects.

We observed a locus-specific ancestry effect at *LPL* such that an increasing amount of European ancestry correlated with higher serum triglycerides (or, equivalently, an increasing amount of African ancestry correlated with lower serum triglycerides). For rs3208305, Africans have more of the allele associated with lower triglycerides, a pattern consistent with the locus-specific ancestry effect. For rs3289 and rs117199990, Africans have more of the allele associated with higher serum triglycerides. Thus, these two associations are inconsistent with the locus-specific ancestry effect. Consequently, the joint test of ancestry and association detected rs3208305 but not rs3289 or rs117199990. Although the associations at rs3289 and rs1177199990 cannot be detected by the joint test, they can be detected using a standard test of association. All three variants have been reported previously to be associated with relevant phenotypes: rs3208305 with postprandial triglyceride levels,[40] rs3289 with triglycerides,[41,42] and rs117199990 with blood lipid metabolites.[43] The previously reported index SNP rs328 (NP_000228.1:p.Ser474Ter), a nonsense variant, was associated with serum triglycerides in unconditional, single-marker analysis.[39] However, after accounting for locus-specific ancestry effects, conditioning on rs3208305 and rs117199990 eliminated all of the association at rs328. Furthermore, genetic scores based on rs3208305, rs3289, and rs117199990 yielded improved prediction of serum triglycerides compared with using just rs117199990, including the prediction of an interethnic difference.

The interval of the locus-specific ancestry effect contains only one gene, *LPL*. We detected no associations at coding variants. The top index variant, rs3208305, tags rs13702 (NC_000008.11:g.19966981T>C), with strong linkage disequilibrium (LD) in both Africans ($r^2 = 0.929$) and European Americans ($r^2 = 0.997$). rs13702 is located in a binding site for miR-410 in the 3′ UTR and is associated with loss of binding.[44,45] We hypothesize that loss of microRNA (miRNA) binding leads to escape from miRNA silencing, higher gene expression, and lower serum triglycerides. The second index variant, rs3289, is directly associated with the gain of a binding site for miR-145 in the 3′ UTR.[44] In this case, we hypothesize that gain of miRNA binding leads to miRNA silencing, lower gene expression, and higher serum triglycerides. The third index variant, rs117199990, tags rs1803924 (NC_000008.11:g.19966163C>T), with strong LD in both Africans ($r^2 = 0.863$) and European Americans ($r^2 = 0.929$). rs1803924 is located in a binding site for miR-579 in the 3′ UTR and is associated with loss of binding.[44,45] As with rs13702, we hypothesize that loss of miRNA binding leads to lower serum triglycerides.

In summary, we have derived a joint test of ancestry and association based on intersection-union testing, in contrast to existing joint tests of ancestry or association based on union-intersection testing. We illustrate the test by examining the association of *LPL* with serum triglycerides in 30,394 African Americans, European Americans, and West Africans. In admixed African Americans, increasing amounts of European ancestry correlated with increased serum triglycerides. Iterative conditional analysis revealed three associated variants, all annotated as eQTLs. The most strongly associated variant, rs3208305, showed allelic differentiation between European Americans and West Africans that was sufficient to explain the locus-specific ancestry effect.

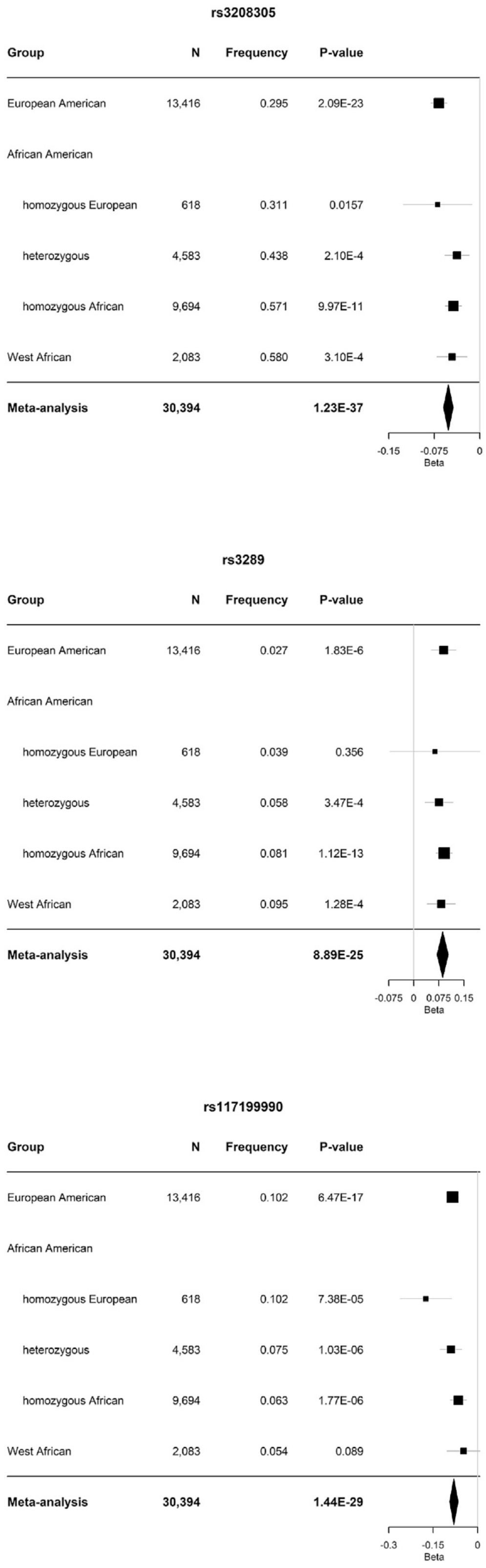

Figure 4. Forest plots depicting the results of the meta-analysis across European Americans, the three ancestral strata of African Americans, and West Africans
"Frequency" indicates the frequency of the alternate allele. Error bars represent 95% confidence intervals.

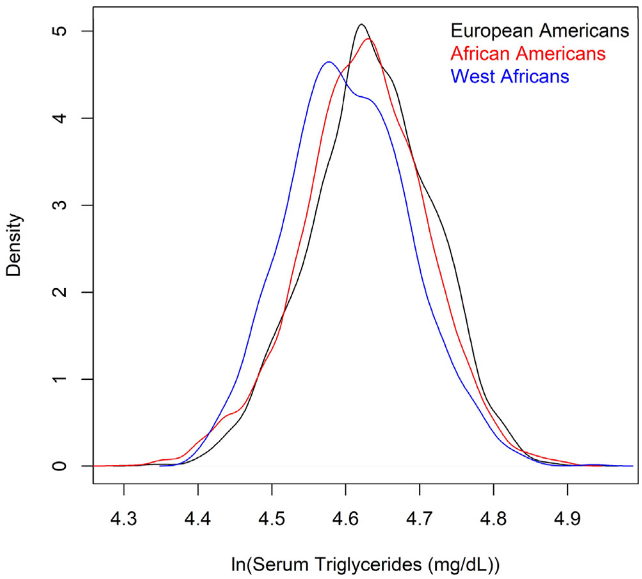

Figure 5. Predicted levels of serum triglycerides
The genetic score accounted for the effects of age, sex, rs3208305, rs3289, and rs117199990.

Controlling for the effects of locus-specific ancestry and multiple variants yielded effect size estimates with no heterogeneity. Collectively, our results underscore the fact that proper analytical methods are more important than millions of samples in order to unlock the power of genetic diversity. Given that populations and individuals of mixed ancestry are the rule, not the exception, our joint test of ancestry and association offers a powerful approach for the analysis of genetically diverse samples with proper control of locus-specific and genome-wide ancestry.

Data and code availability

The datasets used for the analyses in this manuscript were obtained from dbGaP through dbGaP accession study numbers phs000280.v2.p1 (ARIC), phs000285.v3.p2 (CARDIA), phs000284.v1.p1 (CFS), phs000007.v32.p13 (FHS), phs001238.v2.p1 (GENOA), phs000286.v4.p1 (JHS), phs000209.v13.p3 (MESA), phs000433.v1.p1 (SIGNET), and phs000200.v12.p3 (WHI). The HUFS and AADM datasets are available from C.N.R. upon reasonable request.

Supplemental information

Supplemental information can be found online at https://doi.org/10.1016/j.xhgg.2023.100235.

Acknowledgments

This work utilized the computational resources of the NIH HPC Biowulf cluster (https://hpc.nih.gov). The contents of this publication are solely the responsibility of the authors and do not necessarily represent the official view of the National Institutes of

Health. We are thankful to the participants of the AADM project, their families, and their physicians. Support for participant recruitment and initial genetic studies of the parent AADM study was provided by NIH grant 3T37TW00041-03S2 from the Office of Research on Minority Health. The study was supported in part by the Intramural Research Program of the National Institutes of Health in the Center for Research on Genomics and Global Health (CRGGH). The CRGGH is supported by the National Human Genome Research Institute, the National Institute of Diabetes and Digestive and Kidney Diseases, and the Office of the Director at the National Institutes of Health (1ZIAHG200362). The Genotype-Tissue Expression (GTEx) Project was supported by the Common Fund of the Office of the Director of the National Institutes of Health and by NCI, NHGRI, NHLBI, NIDA, NIMH, and NINDS. The data used for the analyses described in this manuscript were obtained from the GTEx Portal (v.8) on March 18, 2022. The funders had no role in the design and conduct of the study; collection, management, analysis, and interpretation of the data; preparation, review, or approval of the manuscript; or decision to submit the manuscript for publication. Acknowledgments and funding for the dbGaP data sets are available in the supplemental information.

Author contributions

D.S. and A.R.B. designed the study, analyzed the data, interpreted the results, and drafted the manuscript; M.H.G., E.F.H., A.P.D., G.C., A.A., and C.N.R. revised the manuscript; J.Z. managed data; and all authors read and approved the final version of the manuscript.

Declaration of interests

C.N.R. is the 2022 President of the American Society of Human Genetics.

Received: December 1, 2022
Accepted: August 28, 2023

Web resources

Bcftools (v.1.9), http://www.htslib.org/download/
Bioconductor package GENESIS (v.2.24.0), https://bioconductor.org/packages/release/bioc/html/GENESIS.html
CheckVCF.py (v.1.4), https://github.com/zhanxw/checkVCF
GCTA (v.1.93.2beta), https://yanglab.westlake.edu.cn/software/gcta/#Download
Liftover, https://genome-store.ucsc.edu/
METAL (v.2018-08-28), https://github.com/statgen/METAL
OMIM: https://www.omim.org/
PLINK (v.1.90b2k), https://www.cog-genomics.org/plink/
R package RMediation (v.1.1.4), https://cran.r-project.org/web/packages/RMediation/index.html
RFMix (v.1.5.4), https://www.dropbox.com/s/cmq4saduh9gozi9/RFMix_v1.5.4.zip
Vcftools (v.0.1.16), https://github.com/vcftools/vcftools

References

1. Rife, D.C. (1954). Populations of hybrid origin as source material for the detection of linkage. Am. J. Hum. Genet. *6*, 26–33.
2. Smith, M.W., Patterson, N., Lautenberger, J.A., Truelove, A.L., McDonald, G.J., Waliszewska, A., Kessing, B.D., Malasky, M.J., Scafe, C., Le, E., et al. (2004). A high-density admixture map for disease gene discovery in African Americans. Am. J. Hum. Genet. *74*, 1001–1013. https://doi.org/10.1086/420856.
3. Patterson, N., Hattangadi, N., Lane, B., Lohmueller, K.E., Hafler, D.A., Oksenberg, J.R., Hauser, S.L., Smith, M.W., O'Brien, S.J., Altshuler, D., et al. (2004). Methods for high-density admixture mapping of disease genes. Am. J. Hum. Genet. *74*, 979–1000. https://doi.org/10.1086/420871.
4. Tishkoff, S.A., Reed, F.A., Friedlaender, F.R., Ehret, C., Ranciaro, A., Froment, A., Hirbo, J.B., Awomoyi, A.A., Bodo, J.-M., Doumbo, O., et al. (2009). The genetic structure and history of Africans and African Americans. Science *324*, 1035–1044. https://doi.org/10.1126/science.1172257.
5. 1000 Genomes Project Consortium, Auton, A., Brooks, L.D., Durbin, R.M., Garrison, E.P., Kang, H.M., Korbel, J.O., Marchini, J.L., McCarthy, S., McVean, G.A., and Abecasis, G.R. (2015). A global reference for human genetic variation. Nature *526*, 68–74. https://doi.org/10.1038/nature15393.
6. Baker, J.L., Rotimi, C.N., and Shriner, D. (2017). Human ancestry correlates with language and reveals that race is not an objective genomic classifier. Sci. Rep. *7*, 1572. https://doi.org/10.1038/s41598-017-01837-7.
7. Dmitrienko, A., Bretz, F., Westfall, P.H., Troendle, J., Wiens, B.L., Tamhane, A.C., and Hsu, J.C. (2010). Multiple Testing Methodology. In Multiple Testing Problems in Pharmaceutical Statistics, A. Dmitrienko, A.C. Tamhane, and F. Bretz, eds. (Chapman & Hall/CRC Press).
8. Erickson, S., Kim, K., and Allison, D.B. (2010). Composite hypothesis testing: an approach built on intersection-union tests and Bayesian posterior probabilities. In Meta-analysis and Combining Information in Genetics and Genomics, R. Guerra and D.R. Goldstein, eds. (Chapman & Hall/CRC Press).
9. Tang, H., Siegmund, D.O., Johnson, N.A., Romieu, I., and London, S.J. (2010). Joint testing of genotype and ancestry association in admixed families. Genet. Epidemiol. *34*, 783–791. https://doi.org/10.1002/gepi.20520.
10. Pasaniuc, B., Zaitlen, N., Lettre, G., Chen, G.K., Tandon, A., Kao, W.H.L., Ruczinski, I., Fornage, M., Siscovick, D.S., Zhu, X., et al. (2011). Enhanced statistical tests for GWAS in admixed populations: assessment using African Americans from CARe and a Breast Cancer Consortium. PLoS Genet. *7*, e1001371. https://doi.org/10.1371/journal.pgen.1001371.
11. Liu, J., Lewinger, J.P., Gilliland, F.D., Gauderman, W.J., and Conti, D.V. (2013). Confounding and heterogeneity in genetic association studies with admixed populations. Am. J. Epidemiol. *177*, 351–360. https://doi.org/10.1093/aje/kws234.
12. Duan, Q., Xu, Z., Raffield, L.M., Chang, S., Wu, D., Lange, E.M., Reiner, A.P., and Li, Y. (2018). A robust and powerful two-step testing procedure for local ancestry adjusted allelic association analysis in admixed populations. Genet. Epidemiol. *42*, 288–302. https://doi.org/10.1002/gepi.22104.
13. Atkinson, E.G., Maihofer, A.X., Kanai, M., Martin, A.R., Karczewski, K.J., Santoro, M.L., Ulirsch, J.C., Kamatani, Y., Okada, Y., Finucane, H.K., et al. (2021). Tractor uses local ancestry to enable the inclusion of admixed individuals in GWAS and to boost power. Nat. Genet. *53*, 195–204. https://doi.org/10.1038/s41588-020-00766-y.
14. Spielman, R.S., McGinnis, R.E., and Ewens, W.J. (1993). Transmission test for linkage disequilibrium: the insulin gene

region and insulin-dependent diabetes mellitus (IDDM). Am. J. Hum. Genet. *52*, 506–516.
15. Hoggart, C.J., Shriver, M.D., Kittles, R.A., Clayton, D.G., and McKeigue, P.M. (2004). Design and analysis of admixture mapping studies. Am. J. Hum. Genet. *74*, 965–978. https://doi.org/10.1086/420855.
16. Montana, G., and Pritchard, J.K. (2004). Statistical tests for admixture mapping with case-control and cases-only data. Am. J. Hum. Genet. *75*, 771–789. https://doi.org/10.1086/425281.
17. Shriner, D., Adeyemo, A., and Rotimi, C.N. (2011). Joint ancestry and association testing in admixed individuals. PLoS Comput. Biol. *7*, e1002325. https://doi.org/10.1371/journal.pcbi.1002325.
18. Teslovich, T.M., Musunuru, K., Smith, A.V., Edmondson, A.C., Stylianou, I.M., Koseki, M., Pirruccello, J.P., Ripatti, S., Chasman, D.I., Willer, C.J., et al. (2010). Biological, clinical and population relevance of 95 loci for blood lipids. Nature *466*, 707–713. https://doi.org/10.1038/nature09270.
19. Bentley, A.R., Chen, G., Shriner, D., Doumatey, A.P., Zhou, J., Huang, H., Mullikin, J.C., Blakesley, R.W., Hansen, N.F., Bouffard, G.G., et al. (2014). Gene-based sequencing identifies lipid-influencing variants with ethnicity-specific effects in African Americans. PLoS Genet. *10*, e1004190. https://doi.org/10.1371/journal.pgen.1004190.
20. Tofighi, D., and MacKinnon, D.P. (2011). RMediation: an R package for mediation analysis confidence intervals. Behav. Res. Methods *43*, 692–700. https://doi.org/10.3758/s13428-011-0076-x.
21. The ARIC Investigators (1989). The Atherosclerosis Risk in Communities (ARIC) Study: design and objectives. Am. J. Epidemiol. *129*, 687–702.
22. Redline, S., Tishler, P.V., Tosteson, T.D., Williamson, J., Kump, K., Browner, I., Ferrette, V., and Krejci, P. (1995). The familial aggregation of obstructive sleep apnea. Am. J. Respir. Crit. Care Med. *151*, 682–687. https://doi.org/10.1164/ajrccm/151.3_Pt_1.682.
23. Daniels, P.R., Kardia, S.L.R., Hanis, C.L., Brown, C.A., Hutchinson, R., Boerwinkle, E., Turner, S.T.; and Genetic Epidemiology Network of Arteriopathy study (2004). Familial aggregation of hypertension treatment and control in the Genetic Epidemiology Network of Arteriopathy (GENOA) study. Am. J. Med. *116*, 676–681. https://doi.org/10.1016/j.amjmed.2003.12.032.
24. Adeyemo, A., Gerry, N., Chen, G., Herbert, A., Doumatey, A., Huang, H., Zhou, J., Lashley, K., Chen, Y., Christman, M., and Rotimi, C. (2009). A genome-wide association study of hypertension and blood pressure in African Americans. PLoS Genet. *5*, e1000564. https://doi.org/10.1371/journal.pgen.1000564.
25. Taylor, H.A., Jr., Wilson, J.G., Jones, D.W., Sarpong, D.F., Srinivasan, A., Garrison, R.J., Nelson, C., and Wyatt, S.B. (2005). Toward resolution of cardiovascular health disparities in African Americans: design and methods of the Jackson Heart Study. Ethn. Dis. *15*. S6–4–17.
26. Bild, D.E., Bluemke, D.A., Burke, G.L., Detrano, R., Diez Roux, A.V., Folsom, A.R., Greenland, P., Jacob, D.R., Jr., Kronmal, R., Liu, K., et al. (2002). Multi-Ethnic Study of Atherosclerosis: objectives and design. Am. J. Epidemiol. *156*, 871–881. https://doi.org/10.1093/aje/kwf113.
27. Garvey, W.T., McLean, D.C., Jr., and Spruill, I. (2003). The Search for Obesity Genes in Isolated Populations: Gullah-Speaking African Americans and the Role of Uncoupling (John Libbey Eurotext), p. 373.
28. Assaf, A.R., and Carleton, R.A. (1994). The Women's Health Initiative Clinical Trial and Observational Study: history and overview. R. I. Med. *77*, 424–427.
29. Friedman, G.D., Cutter, G.R., Donahue, R.P., Hughes, G.H., Hulley, S.B., Jacobs, D.R., Jr., Liu, K., and Savage, P.J. (1988). CARDIA: study design, recruitment, and some characteristics of the examined subjects. J. Clin. Epidemiol. *41*, 1105–1116. https://doi.org/10.1016/0895-4356(88)90080-7.
30. Feinleib, M., Kannel, W.B., Garrison, R.J., McNamara, P.M., and Castelli, W.P. (1975). The Framingham Offspring Study. Design and preliminary data. Prev. Med. *4*, 518–525. https://doi.org/10.1016/0091-7435(75)90037-7.
31. Bentley, A.R., Chen, G., Doumatey, A.P., Shriner, D., Meeks, K.A.C., Gouveia, M.H., Ekoru, K., Zhou, J., Adeyemo, A., and Rotimi, C.N. (2021). GWAS in Africans identifies novel lipids loci and demonstrates heterogenous association within Africa. Hum. Mol. Genet. *30*, 2205–2214. https://doi.org/10.1093/hmg/ddab174.
32. Maples, B.K., Gravel, S., Kenny, E.E., and Bustamante, C.D. (2013). RFMix: a discriminative modeling approach for rapid and robust local-ancestry inference. Am. J. Hum. Genet. *93*, 278–288. https://doi.org/10.1016/j.ajhg.2013.06.020.
33. Liu, Z., Shriner, D., Hansen, N.F., Rotimi, C.N., Mullikin, J.C.; and NISC Comparative Sequencing Program (2020). Admixture mapping identifies genetic regions associated with blood pressure phenotypes in African Americans. PLoS One *15*, e0232048. https://doi.org/10.1371/journal.pone.0232048.
34. Gouveia, M.H., Bentley, A.R., Tarazona-Santos, E., Bustamante, C.D., Adeyemo, A.A., Rotimi, C.N., and Shriner, D. (2022). Unappreciated Subcontinental Admixture in Europeans and European Americans: Implications for Genetic Epidemiology Studies. Preprint at bioRxiv. https://doi.org/10.1101/2022.11.28.518227.
35. Evans, L.M., Tahmasbi, R., Vrieze, S.I., Abecasis, G.R., Das, S., Gazal, S., Bjelland, D.W., de Candia, T.R., Haplotype Reference Consortium, Goddard, M.E., Neale, B.M., et al. (2018). Comparison of methods that use whole genome data to estimate the heritability and genetic architecture of complex traits. Nat. Genet. *50*, 737–745. https://doi.org/10.1038/s41588-018-0108-x.
36. Lettre, G., Lange, C., and Hirschhorn, J.N. (2007). Genetic model testing and statistical power in population-based association studies of quantitative traits. Genet. Epidemiol. *31*, 358–362. https://doi.org/10.1002/gepi.20217.
37. Clarke, G.M., Anderson, C.A., Pettersson, F.H., Cardon, L.R., Morris, A.P., and Zondervan, K.T. (2011). Basic statistical analysis in genetic case-control studies. Nat. Protoc. *6*, 121–133. https://doi.org/10.1038/nprot.2010.182.
38. Howe, K.L., Achuthan, P., Allen, J., Allen, J., Alvarez-Jarreta, J., Amode, M.R., Armean, I.M., Azov, A.G., Bennett, R., Bhai, J., et al. (2021). Ensembl 2021. Nucleic Acids Res. *49*, D884–D891. https://doi.org/10.1093/nar/gkaa942.
39. Graham, S.E., Clarke, S.L., Wu, K.-H.H., Kanoni, S., Zajac, G.J.M., Ramdas, S., Surakka, I., Ntalla, I., Vedantam, S., Winkler, T.W., et al. (2021). The power of genetic diversity in genome-wide association studies of lipids. Nature *600*, 675–679. https://doi.org/10.1038/s41586-021-04064-3.
40. Ibi, D., Noordam, R., van Klinken, J.B., Li-Gao, R., de Mutsert, R., Trompet, S., Christen, T., Blauw, L.L., van Heemst, D., Mook-Kanamori, D.O., et al. (2020). Genome-Wide Association Study of the Postprandial Triglyceride Response Yields Common Genetic

Variation in LIPC (Hepatic Lipase). Circ. Genom. Precis. Med. *13*, e002693. https://doi.org/10.1161/CIRCGEN.119.002693.

41. Gurdasani, D., Carstensen, T., Fatumo, S., Chen, G., Franklin, C.S., Prado-Martinez, J., Bouman, H., Abascal, F., Haber, M., Tachmazidou, I., et al. (2019). Uganda Genome Resource Enables Insights into Population History and Genomic Discovery in Africa. Cell *179*, 984–1002.e36. https://doi.org/10.1016/j.cell.2019.10.004.
42. Noordam, R., Bos, M.M., Wang, H., Winkler, T.W., Bentley, A.R., Kilpeläinen, T.O., de Vries, P.S., Sung, Y.J., Schwander, K., Cade, B.E., et al. (2019). Multi-ancestry sleep-by-SNP interaction analysis in 126,926 individuals reveals lipid loci stratified by sleep duration. Nat. Commun. *10*, 5121. https://doi.org/10.1038/s41467-019-12958-0.
43. Harshfield, E.L., Fauman, E.B., Stacey, D., Paul, D.S., Ziemek, D., Ong, R.M.Y., Danesh, J., Butterworth, A.S., Rasheed, A., Sattar, T., et al. (2021). Genome-wide analysis of blood lipid metabolites in over 5000 South Asians reveals biological insights at cardiometabolic disease loci. BMC Med. *19*, 232. https://doi.org/10.1186/s12916-021-02087-1.
44. Evans, D., Beil, F.U., and Aberle, J. (2013). Resequencing the untranslated regions of the lipoprotein lipase (*LPL*) gene reveals that variants in microRNA target sequences are associated with triglyceride levels. J. Clin. Lipidol. *7*, 610–614. https://doi.org/10.1016/j.jacl.2013.09.006.
45. Caussy, C., Charrière, S., Meirhaeghe, A., Dallongeville, J., Lefai, E., Rome, S., Cuerq, C., Euthine, V., Delay, M., Marmontel, O., et al. (2016). Multiple microRNA regulation of lipoprotein lipase gene abolished by 3'UTR polymorphisms in a triglyceride-lowering haplotype harboring p. Ser474Ter. Atherosclerosis *246*, 280–286. https://doi.org/10.1016/j.atherosclerosis.2016.01.010.

HGGA, Volume *4*

Supplemental information

Universal genome-wide association studies: Powerful joint ancestry and association testing

Daniel Shriner, Amy R. Bentley, Mateus H. Gouveia, Elisabeth F. Heuston, Ayo P. Doumatey, Guanjie Chen, Jie Zhou, Adebowale Adeyemo, and Charles N. Rotimi

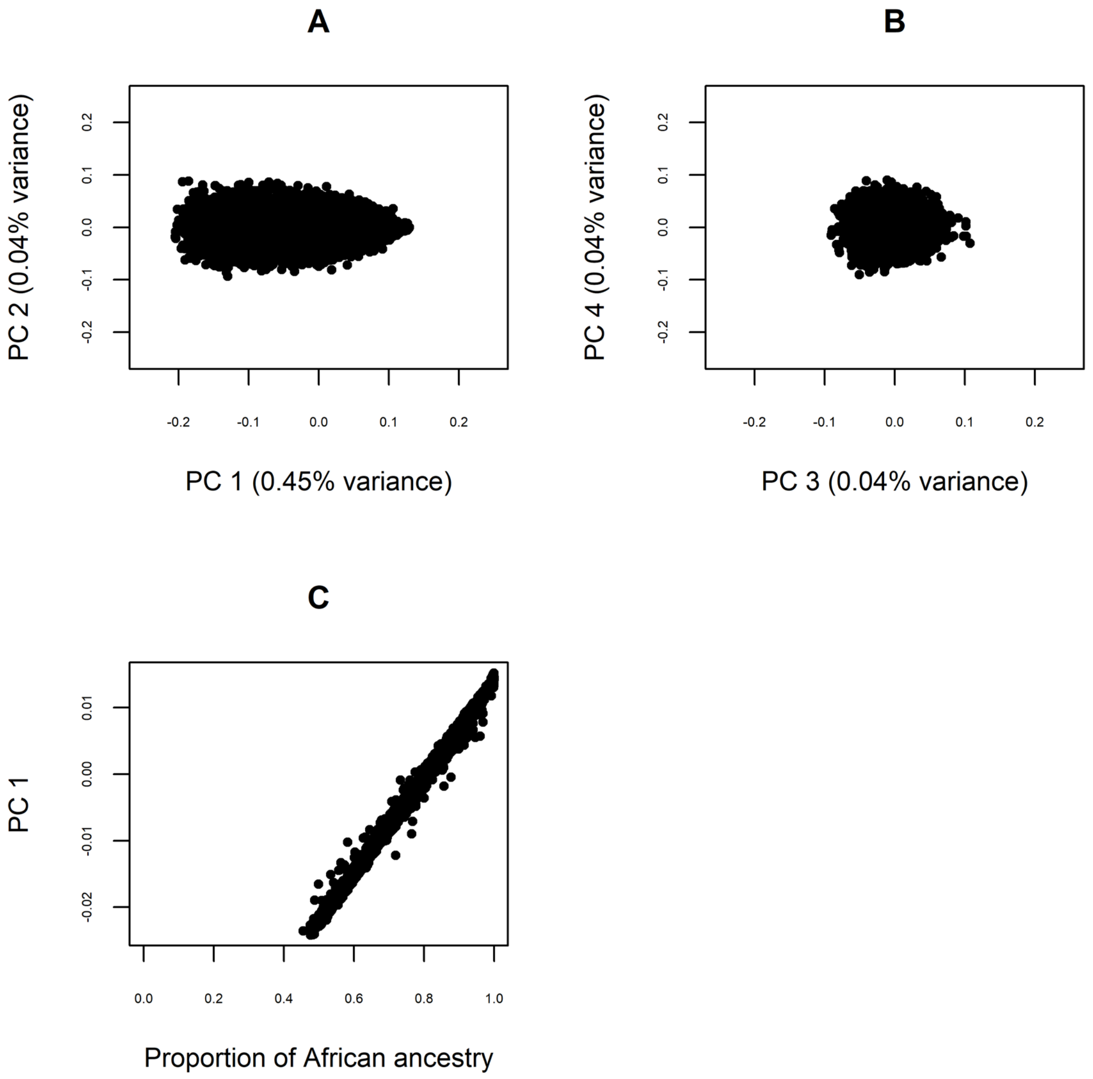

Figure S1. Principal components analysis of the African American data. A) Principal components (PC) 1 and 2. B) Principal components 3 and 4. C) Correlation of the proportion of African ancestry with principal component 1 ($r = 0.998$).

Table S2. Population names and abbreviations.

Population	Code	Metapopulation	Ancestry	Number available	Number included
African Caribbean in Barbados	ACB	AFR	African	96	34
People with African Ancestry in Southwest USA	ASW	AFR	African	61	1
Esan in Nigeria	ESN	AFR	African	99	93
Gambian in Western Division, Mandinka	GWD	AFR	African	113	87
Luhya in Webuye, Kenya	LWK	AFR	African	99	77
Mende in Sierra Leone	MSL	AFR	African	85	76
Yoruba in Ibadan, Nigeria	YRI	AFR	African	108	103
Colombians in Medellín, Colombia	CLM	AMR	Native American	94	0
People with Mexican Ancestry in Los Angeles, CA, USA	MXL	AMR	Native American	64	3
Peruvians in Lima, Peru	PEL	AMR	Native American	85	20
Puerto Ricans in Puerto Rico	PUR	AMR	Native American	104	0
Chinese Dai in Xishuangbanna, China	CDX	EAS	East Asian	93	38
Han Chinese in Beijing, China	CHB	EAS	East Asian	103	85
Southern Han Chinese	CHS	EAS	East Asian	105	97
Japanese in Tokyo, Japan	JPT	EAS	East Asian	104	62
Kinh in Ho Chi Minh City, Vietnam	KHV	EAS	East Asian	99	34
Utah residents (CEPH) with Northern and Western European ancestry	CEU	EUR	European	99	12
Finnish in Finland	FIN	EUR	European	99	31
British in England and Scotland	GBR	EUR	European	91	9
Iberian Populations in Spain	IBS	EUR	European	107	51
Toscani in Italia	TSI	EUR	European	107	33
Bengali in Bangladesh	BEB	SAS	South Asian	86	0
Gujarati Indians in Houston, TX, USA	GIH	SAS	South Asian	103	24
Indian Telugu in the UK	ITU	SAS	South Asian	102	21
Punjabi in Lahore, Pakistan	PJL	SAS	South Asian	96	4
Sri Lankan Tamil in the UK	STU	SAS	South Asian	102	18

Table S3. Reliability of locus-specific ancestry inference.

	Estimated Ancestry				
Reference Panel	African	Native American	East Asian	European	South Asian
AFR	98.96%	0.01%	0.03%	0.95%	0.05%
AMR	0.07%	97.77%	0.00%	2.16%	0.01%
EAS	0.00%	0.04%	99.56%	0.26%	0.13%
EUR	0.32%	0.12%	0.48%	99.06%	0.02%
SAS	0.00%	0.04%	0.14%	0.64%	99.18%

Table S4. Estimated genome-wide ancestral proportions among African Americans.

Study	African*	Native American	East Asian	European	South Asian
ARIC	0.820 [0.001, 1]	0.007 [0, 0.275]	0.007 [0, 0.032]	0.163 [0, 0.985]	0.003 [0, 0.086]
CFS	0.792 [0.288, 0.975]	0.007 [0, 0.246]	0.007 [0, 0.080]	0.191 [0.021, 0.703]	0.003 [0, 0.050]
GENOA	0.826 [0.358, 0.986]	0.007 [0, 0.168]	0.007 [0, 0.032]	0.157 [0.004, 0.618]	0.003 [0, 0.048]
HUFS	0.800 [0, 1]	0.007 [0, 0.449]	0.010 [0, 0.973]	0.180 [0, 0.949]	0.003 [0, 0.277]
JHS	0.822 [0, 0.986]	0.007 [0, 0.118]	0.007 [0, 0.992]	0.161 [0.001, 0.763]	0.003 [0, 0.044]
MESA	0.777 [0.223, 1]	0.007 [0, 0.299]	0.007 [0, 0.501]	0.206 [0, 0.735]	0.003 [0, 0.242]
SIGNET	0.873 [0.001, 1]	0.008 [0, 0.114]	0.004 [0, 0.083]	0.113 [0, 0.992]	0.002 [0, 0.214]
WHI	0.767 [0, 0.999]	0.007 [0, 0.467]	0.007 [0, 0.992]	0.216 [0, 0.994]	0.003 [0, 0.445]

* Mean [minimum, maximum]

Table S10: Expression quantitative trait loci from bulk tissue.

Gencode ID	Gene Symbol	Variant ID	RSID	*P* -Value	Normalized Effect Size	Tissue
ENSG00000175445.14	*LPL*	chr8_19966137_A_T_b38	rs3208305	5.18E-17	0.21	Nerve - Tibial
ENSG00000175445.14	*LPL*	chr8_19966137_A_T_b38	rs3208305	1.31E-07	0.09	Adipose - Subcutaneous
ENSG00000175445.14	*LPL*	chr8_19966137_A_T_b38	rs3208305	3.08E-07	0.22	Whole Blood
ENSG00000175445.14	*LPL*	chr8_19966137_A_T_b38	rs3208305	8.59E-07	0.23	Testis
ENSG00000175445.14	*LPL*	chr8_19966137_A_T_b38	rs3208305	8.94E-07	0.21	Thyroid
ENSG00000175445.14	*LPL*	chr8_19965681_T_C_b38	rs3289	2.32E-12	-0.29	fat
ENSG00000175445.14	*LPL*	chr8_19963405_C_T_b38	rs117199990	1.78E-15	0.56	Whole Blood
ENSG00000175445.14	*LPL*	chr8_19963405_C_T_b38	rs117199990	3.54E-11	2.15	monocyte_CD16_naive
ENSG00000175445.14	*LPL*	chr8_19963405_C_T_b38	rs117199990	7.12E-11	0.86	blood
ENSG00000175445.14	*LPL*	chr8_19963405_C_T_b38	rs117199990	5.02E-08	0.23	Nerve - Tibial
ENSG00000175445.14	*LPL*	chr8_19963405_C_T_b38	rs117199990	2.32E-07	0.74	Spleen

Acknowledgements

The Atherosclerosis Risk in Communities study has been funded in whole or in part with Federal funds from the National Heart, Lung, and Blood Institute, National Institutes of Health, Department of Health and Human Services, under contract numbers HHSN268201700001I, HHSN268201700002I, HHSN268201700003I, HHSN268201700004I, and HHSN268201700005I. The authors thank the staff and participants of the ARIC study for their important contributions. Funding for the GENEVA substudy was provided by National Human Genome Research Institute grant U01HG004402 (E. Boerwinkle). Support for the Coronary Artery Risk Development in Young Adults study was provided by NHLBI grant numbers HHSN268201300025C, HHSN268201300026C, HHSN268201300027C, HHSN268201300028C, and HHSN268201300029C (C. E. Lewis, D. Lloyd-Jones, P. Schreiner, S. Sidney, and J. Shikany). Support for the Cleveland Family Study was provided by NHLBI grant numbers R01 HL46380 and R01 HL113338. The Framingham Heart Study is conducted and supported by the National Heart, Lung, and Blood Institute (NHLBI) in collaboration with Boston University (contracts N01-HC-25195, HHSN268201500001I, and 75N92019D00031). This manuscript was not prepared in collaboration with investigators of the Framingham Heart Study and does not necessarily reflect the opinions or views of the Framingham Heart Study, Boston University, or NHLBI. Funding to support the Omni cohort recruitment, retention, and examination was provided by NHLBI contracts N01-HC-25195, HHSN268201500001I, and 75N92019D00031, as well as NHLBI grants R01-HL070100, R01-HL076784, R01-HL49869, and U01-HL-053941. SHARe Illumina genotyping was provided under an agreement between Illumina and Boston University. Support for GENOA was provided by the National Heart, Lung and Blood Institute (HL054457, HL054464, HL054481, HL119443, and HL087660) of the

National Institutes of Health. We would like the thank the Mayo Clinic Genotyping Core, the DNA Sequencing and Gene Analysis Center at the University of Washington, and the Broad Institute for their genotyping and sequencing services. We would like to thank the GENOA participants. This manuscript was not prepared in collaboration with investigators from the Genetic Epidemiology Network of Arteriopathy and does not necessarily reflect the opinions or views of the Genetic Epidemiology Network of Arteriopathy or NHLBI. The Jackson Heart Study (JHS) is supported and conducted in collaboration with Jackson State University (HHSN268201800013I), Tougaloo College (HHSN268201800014I), the Mississippi State Department of Health (HHSN268201800015I/HHSN26800001) and the University of Mississippi Medical Center (HHSN268201800010I, HHSN268201800011I and HHSN268201800012I) contracts from the National Heart, Lung, and Blood Institute (NHLBI) and the National Institute for Minority Health and Health Disparities (NIMHD). The authors wish to thank the staffs and participants of the JHS. Funding for CARe genotyping was provided by NHLBI Contract N01-HC-65226. MESA and the MESA SHARe project are conducted and supported by the National Heart, Lung, and Blood Institute (NHLBI) in collaboration with MESA investigators. Support for MESA is provided by contracts N01-HC95159, N01-HC-95160, N01-HC-95161, N01-HC-95162, N01-HC-95163, N01-HC-95164, N01-HC-95165, N01-HC95166, N01-HC-95167, N01-HC-95168, N01-HC-95169, UL1-RR-025005, and UL1-TR-000040. Funding for SHARe genotyping was provided by NHLBI Contract N02-HL-64278. Genotyping was performed at Affymetrix (Santa Clara, California, USA) and the Broad Institute of Harvard and MIT (Boston, Massachusetts, USA) using the Affymetrix Genome-Wide Human SNP Array 6.0. This manuscript was not prepared in collaboration with MESA investigators and does not necessarily reflect the opinions or views of MESA, or the NHLBI. Support for SIGNET

is provided by R01-DK084350 (M. M. Sale), U01-NS041588 (G. Howard), M01-RR001070 (J. K. Fernandes), P20-RR017696 (K. L. Kirkwood), and P60-AR049459 (G. S. Gilkeson) and by the W. M. Keck Foundation (W. T. Garvey). The WHI program is funded by the National Heart, Lung, and Blood Institute, National Institutes of Health, U.S. Department of Health and Human Services through contracts HHSN268201600018C, HHSN268201600001C, HHSN268201600002C, HHSN268201600003C, and HHSN268201600004C. This manuscript was not prepared in collaboration with investigators of the WHI, has not been reviewed and/or approved by the Women's Health Initiative (WHI), and does not necessarily reflect the opinions of the WHI investigators or the NHLBI. Funding for WHI SHARe genotyping was provided by NHLBI contract N02-HL-64278.
